# Predictors of Treatment Success of Psychotherapy in Functional Disorders: A Systematic Review of the Literature

**DOI:** 10.1002/cpp.70075

**Published:** 2025-04-23

**Authors:** Caroline Rometsch, Alexandra Martin, Fiammetta Cosci

**Affiliations:** ^1^ Department of Experimental and Clinical Medicine University of Florence Italy; ^2^ School of Human and Social Sciences University of Wuppertal Wuppertal Germany; ^3^ Department of Health Sciences University of Florence Italy; ^4^ Department of Psychiatry and Neuropsychology Maastricht University Netherlands

**Keywords:** functional disorders, predictor, psychotherapy, systematic review, treatment success

## Abstract

**Objective:**

Functional disorders (FDs) benefit from psychotherapy. However, the determinants predicting their efficacy remain largely unexplored.

**Methods:**

A systematic literature review was conducted. PubMed, Web of Science, Embase, Cochrane collaboration and grey literature were screened from inception to November 2024. Randomized controlled trials on predictors of success of psychotherapy for FDs (e.g., somatoform disorders, irritable bowel syndrome [IBS], chronic fatigue syndrome/myalgic encephalomyelitis [CFS/ME], fibromyalgia [FM]) in adults (i.e., ≥ 18 years of age) were included. The review yielded 24 eligible studies and included 3382 participants. A standardized quality assessment via ROB‐2 Tool was performed. PRISMA guidelines were followed.

**Results:**

Most studies applied CBT‐based interventions (*n* = 19), mainly face‐to‐face, with some internet‐based (*n* = 5), while fewer used emotional‐based (*n* = 4), mindfulness‐based (*n* = 3), psychodynamic (*n* = 1) or operant behavioural therapy (*n* = 1). The primary factors identified as predictive of treatment success in FM and somatization were the intensity of experienced pain. Moreover, the presence of mental disorders, i.e., depression and anxiety disorders, emerged as predictors for a range of disorders including FM, IBS, somatization disorder, hypochondriasis, medically unexplained symptoms and dissociative seizures. Symptom severity was recognized as a predictor across various FDs with findings indicated that severe severity could predict treatment outcomes.

**Conclusion:**

The body of research concerning predictors of treatment success in the context of FDs can help clinicians identifying appropriate psychotherapy trajectories.

**Trial Registration:** Not applicable. PROSPERO no. CRD42022379791; OSF (https://osf.io/8q7z9).

## 1 Introduction

Functional disorders (FDs) encompass a broad spectrum of persistent somatic symptoms with various aetio‐pathogenetic explaining models (Saunders et al. 2023), characteristically observed in a range of psychosomatic disorders, including but not limited to fibromyalgia (FM), irritable bowel syndrome (IBS), hypochondriasis and chronic fatigue syndrome (CFS) (Burton et al. 2020). FDs have an estimated point prevalence of around 9% among the adult European general population (Rometsch et al. 2024), are associated with substantial healthcare utilization (Chaabouni et al. 2023) and significant health‐related costs (Lagrand et al. 2023). This is partly attributed to prevalent mental disorder comorbidities, where anxiety and depressive disorders are the most common (De Waal et al. 2004; Carle‐Toulemonde et al. 2023). The diagnosis of FD may vary according to the standardized nosographic system used, which include, among the others, the Diagnostic and Statistical Manual of Mental Disorders (DSM) (Association 2013), the International Classification of Diseases (ICD) (World Health Organization 1992) and the Diagnostic Criteria for Psychosomatic Research (DCPR) (Fava et al. 1995; Fava et al. 2017), in their various editions. Syndrome‐specific standardized diagnostic systems also allow to formulate diagnoses, for instance, the American College of Rheumatology criteria (ACR) are specific for FM while the Rome criteria are specific for IBS.

Pharmacological treatment used for FD, such as for instance antidepressants, showed low or no evidence of effectiveness or efficacy (Kleinstaeuber et al. 2014). Given these limitations, non‐pharmacological approaches have become increasingly important. Among them, psychotherapy, particularly Cognitive Behavioural Therapy (CBT) (Liu et al. 2019) and short‐term psychodynamic psychotherapy (Abbass et al. 2021), showed from moderate to large sustained benefits in patients with functional somatic symptoms. Less structured psychological interventions (e.g., relaxation therapy, hypnotherapy) have been proposed (Ford et al. 2014), but showed limited utility. Multi‐component approaches seem promising (Drossman and Thompson 1992; Häuser et al. 2009; Kustra‐Mulder et al. 2023; Mamo et al. 2023).

Of course, the effect of an intervention is related to several ingredients and for this reason, it needs to be evaluated based on primary/secondary outcomes. A reflection on outcome predicting factors is needed to translate the results of research into the clinical realm and let the scientific literature have a clinical impact. Identifying such predictors can inform treatment personalization, help clinicians tailor interventions and refine therapeutic strategies. For instance, clinical characteristics, such as more severe symptoms, longer symptom duration, lower physical/emotional/social functioning, were found to be associated with poorer outcome when CBT was administered to patients with medically unexplained symptoms (MUS) (Sarter et al. 2022). Sex showed to moderate the effects of integrative group psychotherapy in functional vertigo (Dale et al. 2023).

Since the generalizability across the FD spectrum remains unclear and a comprehensive synthesis of predictors across different psychotherapeutic approaches for FDs is lacking, the present study aimed at systematically reviewing the literature on such predictors in adults of the general population with FDs across different psychotherapeutic interventions.

## 2 Methods

### 2.1 Eligibility Criteria

Articles published in peer‐reviewed journals reporting on predictors of success of psychotherapy for FDs in adults (i.e., ≥ 18 years of age) were included. Studies with a randomized‐controlled study design were selected to guarantee a rigorous scientific methodology. Psychotherapy included psychodynamic therapy, CBT in various versions (e.g., Mindfulness‐Based Stress Reduction [MBSR], Acceptance and Commitment Therapy [ACT], eclectic therapies, emotional awareness and expression therapy). Possible comparators were waiting list, placebo, clinical management/treatment as usual or enhanced care. A minimum of four sessions delivered via a direct contact (either in person or online) of the active treatment was required (Flückiger et al. 2020) in order to be sure that at least part of the treatment was delivered. Research on in‐ or out‐patients was considered eligible. FDs included somatoform disorders (also named somatization disorder, somatic symptom disorder, bodily distress disorder), IBS, CFS/myalgic encephalomyelitis (ME), FM, dissociative seizures, functional gastrointestinal symptoms. FDs had to be diagnosed using a standardized clinical taxonomy (i.e., DSM, ICD, DCPR, ACR‐1990, Rome criteria, U.S. Centers for Disease Control criteria) by a professional (either clinician or researcher) or via a diagnostic tool (e.g., endoscopy). No restriction to language or country was applied. For eligibility, PICOS criteria (Methley et al. 2014) were followed (see Table S2 online supplementary material).

### 2.2 Outcomes

Predictors of primary outcomes were symptom intensity/severity, number of symptoms. Secondary outcomes were the following: clinical manifestations related to FD (e.g., fatigue); functional disability (e.g., functional limitations); quality of life; well‐being; severity of mental comorbidities (i.e., anxiety/depression disorders) (Friedman et al. 2015); psychotherapy‐related issues (e.g., treatment expectations/motivation) (Gergov et al. 2021); others (e.g., drugs, hospitalization duration). Even though not modifiable, sociodemographic predictors were considered (i.e., age, sex, socioeconomic status [SES], employment).

### 2.3 Information Sources and Search Strategy

PubMed, Web of Science, Embase and the Cochrane Library were systematically searched from inception to November 2024 applying the search terms referring to FDs (see supplementary material) combined via the Boolean ‘AND’ operator with “psychotherapy” ‘AND’ “efficacy OR effective* OR benefit OR predict*” ‘AND’ ((RCT) OR (“randomized controlled trial*”)) OR (“randomised controlled trial*”)) (see Table S3). A manual search of reference lists and grey literature (i.e., Opengrey) was conducted. Duplicates were identified and articles systematically screened via the open‐source online tool Rayyan (Ouzzani et al. 2016). The Preferred Reporting Items for Systematic Reviews and Meta‐analysis (PRISMA) guidelines (Page et al. 2021) were followed. Two independent reviewers (CR and FC) conducted a parallel screening of potential articles and evaluated full texts. In case of disagreement, a third reviewer (AM) was enlisted to arbitrate and achieve consensus. The study protocol was preregistered on PROSPERO no. CRD42022379791 and on OSF (https://osf.io/8q7z9).

### 2.4 Data Extraction and Quality Assessment

A standardized data collection form was developed to collect information on population, sample size, age, sex, drop‐outs, characteristics of the experimental intervention, number and duration of sessions, comparators, assessment tools, primary outcomes (reported as standardized mean differences [Cohen's d], interaction terms, coefficient of determination [*R* ^*2*^], correlation coefficient [r], regression coefficient [B], standard error [SE], predicted group membership, canonical correlation, direct and indirect effects with 95%CI, regression coefficient with incidence rate ratios, and standardized path coefficients), secondary outcomes (reported as number needed to treat [NNT], interaction coefficient and odds ratios [ORs], regression coefficients, Group × Time Interaction, correlations, indirect effects in path analyses, bias‐corrected bootstrapped indirect effects, maximum likelihood estimation, time effects, treatment effects, predicted group membership, standardized path coefficients, interaction terms and coefficient of determination). The quality of the studies was independently assessed by CR and FM using ROB2 Tool revealing a low risk of bias, ensuring a high level of confidence in the integrity and reliability of the findings, which resulted throughout in a low judgement besides some concerns regarding one study (Vallejo et al. 2015).

## 3 Results

A total of 44,367 articles were retrieved, complemented by 28 articles identified through manual check of reference lists. A total of 296 articles were deemed suitable for an in‐depth full‐text review. Among them, 24 met the inclusion criteria (see Table S1 in the online supplementary material) encompassing 3382 participants and pertained to the following diagnoses: FM (*n* = 5), IBS (*n* = 5), hypochondriasis (*n* = 4), CFS (*n* = 4), MUS (*n* = 3), somatization (*n* = 1), dissociative seizures (*n* = 1) and functional dyspepsia (*n* = 1). Data on predictors of treatment success were categorized according to predicting primary (see Table 1) and secondary outcomes (see also Table 2).

**TABLE 1:** Classification of identified primary predictors for treatment success of functional disorders.

Author	Diagnosis	Outcome variable	Predictors referring to sociodemo‐graphic variables	Predictors referring to mental comorbidities	Predictors referring to FD symptoms	Predictors referring to physical condition	Predictors referring to emotions, cognitions, behaviour	Predictors referring to applied treatments (pharmacotherapy)	Predictors referring to treatment‐related factors
Andrés‐Rodríguez et al. (2019)	FM	Symptom severity				Pro‐inflammatory markers			
Friesen et al. (2017)	FM	Symptom severity			Higher symptom severity				
Karlsson et al. (2015)	FM	Symptom severity			Baseline levels of pain severity, Pain duration, number of tender‐points				
Thieme et al. (2007)	FM	Symptom severity			Pain symptoms	Functional impairment			
Guthrie et al. (1991)	IBS	Symptom severity		Anxiety and depression					
Henrich et al. (2020)	IBS	Symptom severity		Anxiety	Not‐significant: Pain catastrophizing				
Lackner et al. (2010)	IBS	Higher symptom severity							
Ljótsson et al. (2011)	IBS		No predictor identified						
Maroti et al. (2022)	Somatic symptom disorder	Pain intensity		Depression (mediator)					
Heins et al. (2010)	CFS	Symptom severity				Functional impairment			
Prins et al. (2001)	CFS	Symptom severity		Higher baseline symptom severity	Focus on bodily symptoms	Passive activity	Sense of control		
Tummers et al. (2010)	CFS	Symptom severity			Fatigue‐related symptoms				Guided self‐instruction
Kolk et al. (2004)	MUS	Symptom severity	Not‐significant: Age, gender, socioeconomic status, employment status, housekeeping status		Less somatic attribution	Not‐significant: Chronic diseases	Negative affectivity, Not‐significant: Selective attention		
Axelsson, Andersson, et al. (2020)		Hypochondriasis‐related behaviours					Emotional detachment in stressful situations Not‐significant: Baseline levels of non‐reactivity		Not‐significant: Perceived competence
Nakao et al. 2011)	Hypochondrias	Symptom severity	Marriage		Pretreatment anxiety				
Richtberg et al. (2017)	Hypochondrias	Symptom severity	Not‐significant: Sociodemographic variables general psychopathology	Mental comorbidities: Obsessive‐compulsive disorder	Pretreatment levels of hypochondriasis				Interpersonal therapy dynamics
Weck et al. (2015)	Hypochondrias	Symptom severity			Not‐significant: Symptom report, categorizing health‐related norms		Illness‐related evaluation of bodily symptoms (mediator)		
Goldstein et al. (2022)	Dissociative seizures	Dissociative seizures frequency at 12‐months	Not‐significant: Sex	Not‐significant: Mental comorbidity	Not‐significant: Symptom severity				
Haug et al. (1994)	Functional dyspepsia	No predictors identified							

*Note:* CFS = Chronic fatigue syndrome, FM = Fibromyalgia, IBS = Irritable bowel syndrome, MUS = medically unexplained symptoms.

**TABLE 2:** Classification of identified secondary predictors for treatment success of functional disorders.

Author	Diagnosis	Outcome variable	Predictors referring to sociodemo‐graphic variables	Predictors referring to mental comorbidities	Predictors referring to FD symptoms	Predictors referring to physical condition	Predictors referring to emotions, cognitions, behaviour	Predictors referring to applied treatments (pharmacotherapy)	Predictors referring to treatment‐related factors
Andrés‐Rodríguez et al. (2019)	FM	Anxiety and depression, mindfulness and psychological flexibility			Symptom severity				
Friesen et al. (2017)	FM	Improvements in quality of life, fear of movement, and fatigue		Anxiety/depression			Psychological and emotional baseline status		
Karlsson et al. (2015)	FM	Improvements in life control, affective distress, and depression, perceived social support, vital exhaustion	Employment status	Stress and exhaustion levels			Perceptions of support		
Vallejo et al. (2015)	FM	Psychological distress, fatigue					Catastrophizing and coping strategies (e.g., relaxation, self‐efficacy)		
Blanchard et al. (2006)	IBS	Quality of life, psychological distress		Higher baseline trait anxiety	Baseline diarrhoea severity				
Guthrie et al. (1991)	IBS	Symptom severity			Symptom duration, limited number of body areas, stress‐induced pain				
Lackner et al. (2010)	IBS	Treatment response	Female sex						
Ljótsson et al. (2011)	IBS	Symptom severity	No predictor identified						
Heins et al. (2010)	CFS	Reduction in pain, functional impairment, and psychological distress				Baseline levels of functional impairment			
Goedendorp et al. (2013)	CFS	Increased dropout rates and persistent functional impairments					Mnestic difficulties (e.g., memory and neuropsychological tests)		
Prins et al. (2001)	CFS	Functional impairment			Focus on bodily symptoms	Baseline functional impairment			
Tummers et al. (2010)	CFS	Physical functioning			Fatigue‐related symptoms				Treatment condition
Van Ravesteijn et al. (2013)	MUS	Physical functioning	Older age						
Zonneveld et al. (2012)	MUS	Quality of life	Better mental health‐related quality of life	Psychological symptoms, psychiatric history			Fewer personality‐disorder characteristics		
Kolk et al. (2004)	MUS	Negative affectivity, psychological attribution, anxiety							Eclectic therapy
Goldstein et al. (2022)	Dissociative seizures	Quality of life, reduction in work and social adjustment	Age, Occupation/Education, Employment benefits status	Depression/anxiety	Duration since symptom onset, symptom count				Confidence in diagnosis, belief in therapy effectiveness
Haug et al. (1994)	Functional dyspepsia	No predictors identified							

*Note:* CFS = Chronic fatigue syndrome, FM = Fibromyalgia, IBS = Irritable bowel syndrome, MUS = medically unexplained symptoms.

### 3.1 Fibromyalgia

Five studies pertained to FM (Thieme et al. 2007; Karlsson et al. 2015; Vallejo et al. 2015; Friesen et al. 2017; Andrés‐Rodríguez et al. 2019). The studies enrolled females in most studies besides in Friesen et al. (2017) where over 80% were females; with an average age from 45 (Thieme et al. 2007) to 53 years (Vallejo et al. 2015; Andrés‐Rodríguez et al. 2019). The studies encompassed sample sizes ranging from 20 (Vallejo et al. 2015) to 140 (Vallejo et al. 2015). Two studies were conducted in Spain (Vallejo et al. 2015, Andrés‐Rodríguez et al. 2019), while Germany (Thieme et al. 2007), Sweden (Karlsson et al. 2015) and Canada (Friesen et al. 2017) contributed with one study. Four studies employed CBT (Thieme et al. 2007; Karlsson et al. 2015; Vallejo et al. 2015; Friesen et al. 2017), which was internet‐based in Vallejo et al. (2015) and Friesen et al. (2017), in a group format in (Karlsson et al. 2015). One study implemented mindfulness‐based stress reduction (Andrés‐Rodríguez et al. 2019). The total number of therapeutic sessions varied from 5 (Friesen et al. 2017), and 8 (Andrés‐Rodríguez et al. 2019) to 15 (Thieme et al. 2007), with each session lasting between 120 (Thieme et al. 2007; Vallejo et al. 2015; Friesen et al. 2017; Andrés‐Rodríguez et al. 2019) and 180 min (Karlsson et al. 2015).

#### 3.1.1 Predicting Primary Outcomes

A predictor for improvements in symptom severity was baseline severity of FM symptoms. Those with more severe initial symptoms generally showed greater improvements compared to the waiting list (Friesen et al. 2017). Baseline levels of pain severity were significant predictors of symptom severity after CBT intervention. Additionally, duration of generalized pain, number of tender points, co‐morbidities as well as drugs use (e.g., analgesics and antidepressants) were evaluated as potential covariates, but these variables were not explicitly identified as significant predictors of treatment outcome in this study (Karlsson et al. 2015). Pain decreased in patients with lower physical impairment before treatment when psychological pain treatment strategies were implemented, as compared to those with higher physical impairment who did not show similar levels of improvement (Thieme et al. 2007). Higher baseline levels of pro‐inflammatory markers predicted greater symptom severity and were predictors of less improvement in psychological inflexibility following MBSR (Andrés‐Rodríguez et al. 2019).

#### 3.1.2 Predicting Secondary Outcomes

Patients receiving MBSR plus treatment as usual (TAU), as opposed to MT plus TAU or TAU alone, showed significant reduction in anxiety and depression, as well as improvement in mindfulness and psychological flexibility compared to TAU (Andrés‐Rodríguez et al. 2019). When internet‐delivered CBT (iCBT), face‐to‐face CBT and waitlist (WL) were compared, patients who received iCBT and CBT exhibited lower psychological distress (i.e., depression, catastrophizing thoughts, rumination, helplessness behaviour) and used relaxation techniques more effectively, resulting in lower fatigue, particularly under iCBT (Vallejo et al. 2015). Improvements in quality of life, fear of movement and fatigue were predicted by the psychological and emotional baseline status. Participants with higher initial anxiety or depression reported significant improvement after the internet‐delivered CBT intervention compared to controls (Friesen et al. 2017). Initial symptom severity served as a significant predictor for improvements in life control, affective distress and depression (Karlsson et al. 2015). Initial perceptions of support significantly predicted improvements in perceived support from spouses or significant others (Karlsson et al. 2015). Furthermore, baseline stress and exhaustion levels were significant predictors for reductions in vital exhaustion and stress behaviour over time (Karlsson et al. 2015). Employment status was a significant predictor for enhanced life control (Karlsson et al. 2015).

### 3.2 Irritable Bowel Syndrome

Five studies focused on IBS (Guthrie et al. 1991; Blanchard et al. 2006; Lackner et al. 2010; Ljótsson et al. 2011; Henrich et al. 2020). The majority of participants were female, ranging from 74% (Ljótsson et al. 2011) to 100% of the samples (Lackner et al. 2010; Henrich et al. 2020). The average age spanned from 33.5 (Ljótsson et al. 2011) to 49 years (Blanchard et al. 2006). The sample size was from 23 (Ljótsson et al. 2011, Henrich et al. 2020) to 151 patients (Blanchard et al. 2006). Two studies (Guthrie et al. 1991; Henrich et al. 2020) were conducted in Great Britain, two in the USA (Blanchard et al. 2006; Lackner et al. 2010) and one in Sweden (Ljótsson et al. 2011). In one study, a psychodynamic intervention of seven sessions lasting 120 min each (Guthrie et al. 1991) was administered compared to waiting list. In two studies (Ljótsson et al. 2011, Henrich et al. 2020), CBT‐based interventions offering six sessions of 120 min each (Henrich et al. 2020) or 10 sessions of 45 min each (Ljótsson et al. 2011) versus waiting list were implemented. One study used CBT against psychoeducational support in 10 weekly sessions (Blanchard et al. 2006) or against waiting list (Lackner et al. 2010). Only one study used iCBT (Ljótsson et al. 2011).

#### 3.2.1 Predicting Primary Outcomes

The decrease of symptom severity was predicted by a diagnosis of anxiety or depression, which was observed for all patients undergoing the psychodynamic treatment compared to those in the waiting list (Guthrie et al. 1991). The MBCT group experienced significant reduction in IBS symptoms compared to the waiting list. Anxiety was found to be a mediator for the impact of MBCT on symptom severity. Pain catastrophizing did not significantly explain the effect of mindfulness treatment on IBS symptom severity as a mediator (Henrich et al. 2020) and further did not significantly predict a decrease in IBS symptoms under iCBT (Ljótsson et al. 2011). Greater baseline IBS symptom severity predicted a more substantial reduction in IBS symptoms among those who received CBT (Lackner et al. 2010).

#### 3.2.2 Predicting Secondary Outcomes

When pain is increasing under stress, episodic or variable, of brief duration, or in a limited number of body areas, it was a predictor of treatment success in patients undergoing psychodynamic therapy (Guthrie et al. 1991). Baseline diarrhoea severity predicted improvements in IBS‐specific quality of life and higher baseline trait anxiety was a predictor of poorer psychological distress outcomes (Blanchard et al. 2006). A predictor for rapid treatment response to CBT in IBS was female sex (Lackner et al. 2010).

### 3.3 Hypochondriasis

Four studies (Nakao et al. 2011; Weck et al. 2015; Richtberg et al. 2017; Axelsson, Hesser, et al. 2020) were conducted applying CBT for hypochondriasis/health anxiety. One study used 12 sessions of internet‐delivered CBT versus waiting list on 204 participants (70% females, mean age 39 years) and was conducted in Sweden (Axelsson, Hesser, et al. 2020). An internet‐based CBT randomized controlled trial in cross‐over‐design with another 12 weekly sessions was conducted in Sweden on 81 participants (74% female, mean age of 39.0 years). In the USA, a CBT intervention versus usual medical care was conducted on 182 patients (76% females, mean age 42.1 years) (Nakao et al. 2011). Two German studies focused on hypochondriasis referring to the same sample, which comprised 75 patients (59% females) having an average age of 43 years (Weck et al. 2015; Richtberg et al. 2017). Exposure therapy (ET) and cognitive therapy (CT) were compared to waiting list in 21 patients who received 12 individual sessions of 50 min each (Weck et al. 2015, Richtberg et al. 2017).

#### 3.3.1 Predicting Primary Outcomes

CBT demonstrated efficacy in reducing hypochondriasis‐related behaviours and enhancing non‐reactivity and perceived competence compared to waiting list. While improvements were predicted by greater increases in non‐reactivity (e.g., emotional detachment in stressful situations) and perceived competence during therapy, baseline levels of non‐reactivity and perceived competence (e.g., confidence in managing health concerns) did not predict reductions in symptom severity of hypochondriasis (Axelsson, Hesser, et al. 2020). High pretreatment anxiety predicted greater reductions in symptom severity of hypochondriasis (Nakao et al. 2011). Reduction/change in hypochondriacal symptoms was observed under CT and ET without differences among the two and waiting list (Richtberg et al. 2017). Pretreatment levels of hypochondriasis were significant predictors of posttreatment symptom severity. Comorbid mental disorders were significant predictors of symptom severity at 12‐month follow‐up. Patients' interpersonal behaviour during the sessions was a significant predictor of hypochondriasis symptom decrease; however, the general psychopathology and sociodemographic factors were shown to be not significant (Richtberg et al. 2017). Understanding and addressing how patients perceive and interpret their personal health concerns, as opposed to evaluating symptoms in other people or in a general context, was found to be a crucial mediator in the relationship between CT/ET and hypochondriasis severity decrease (Weck et al. 2015). Symptom report and categorizing health‐related norms did not significantly mediate the relationship between CT/ET and hypochondriasis severity decrease (Weck et al. 2015). Being married was a significant predictor of reduction in somatic symptoms in those who received CBT, even after controlling for baseline depression and other demographic factors (Nakao et al. 2011).

### 3.4 Chronic Fatigue Syndrome

Four studies investigated CFS including patients with an average age from 37 (Heins et al. 2010) (about two‐thirds were females) to 49.5 years (Andrés‐Rodríguez et al. 2019). All studies were conducted in The Netherlands (Prins et al. 2001; Heins et al. 2010; Tummers et al. 2010; Goedendorp et al. 2013). CBT interventions were implemented versus normal care in 508 patients (Heins et al. 2010) and in 85 participants (Tummers et al. 2010) while another study used guided CBT‐intervention in 278 patients (Prins et al. 2001), both against waiting list, and in a latter one CBT was implemented in 171 patients (Goedendorp et al. 2013).

#### 3.4.1 Predicting Primary Outcomes

Greater baseline sense of control predicted better improvement in fatigue severity, while a passive activity pattern and higher focus on bodily symptoms were associated with poorer outcomes. Higher baseline fatigue severity predicted less improvement in fatigue (Prins et al. 2001). A reduction in fatigue was observed in both the stepped care and care‐as‐usual conditions; only after guided self‐instruction, the proportion of patients experiencing a clinically significant improvement was lower compared to both stepped care and care as usual (Tummers et al. 2010). High baseline levels of functional impairment predicted fatigue deterioration particularly in those who received CBT (Heins et al. 2010).

#### 3.4.2 Predicting Secondary Outcomes

An increase in physical functioning was achieved in CBT and waiting list (Tummers et al. 2010). Reduction in pain, functional impairment and psychological distress were predicted by high baseline levels of functional impairment (Heins et al. 2010). A predictor for increased dropout rates and persistent functional impairments following CBT was underperformance (e.g., memory and neuropsychological tests) (Goedendorp et al. 2013). For functional impairment, higher baseline impairment scores and greater focus on bodily symptoms predicted smaller improvements (Prins et al. 2001).

### 3.5 Medically Unexplained Symptoms

MUS were investigated (Kolk et al. 2004; Van Ravesteijn et al. 2013) in three studies with up to 162 patients (Zonneveld et al. 2012) having an average age from 35 (Kolk et al. 2004) to 47 years (Van Ravesteijn et al. 2013). Approximately two‐thirds of participants were females (Kolk et al. 2004, Van Ravesteijn et al. 2013). The studies were conducted in The Netherlands (Kolk et al. 2004; Zonneveld et al. 2012; Van Ravesteijn et al. 2013). In one study, psychotherapy was provided in individual sessions, which varied depending on the therapists' orientation and included cognitive–behavioural, client‐centred or eclectic therapies vs. TAU (Kolk et al. 2004). Zonneveld et al. (2012) applied 13 sessions of CBT consisting of 13 weekly 2‐h group sessions versus waiting list (Zonneveld et al. 2012). One study compared mindfulness‐based cognitive therapy plus enhanced usual care (EUC) and EUC alone (Van Ravesteijn et al. 2013). The MBCT plus EUC group included 80 patients who received eight sessions of 150 min each (Van Ravesteijn et al. 2013).

#### 3.5.1 Predicting Primary Outcomes

Symptom reduction of self‐reported unexplained symptoms was predicted by negative affectivity and less somatic attribution and was mediated by psychological attribution and pre‐treatment anxiety. Symptom reduction was similar in both EUC and TAU conditions (Kolk et al. 2004). Age, sex, socioeconomic status, chronic diseases, employment status, housekeeping status and selective attention did not significantly predict symptom severity (Kolk et al. 2004).

#### 3.5.2 Predicting Secondary Outcomes

Predictors for short‐term improvement in physical quality of life included more psychological symptoms, fewer personality‐disorder characteristics, the presence of a psychiatric history and better mental health‐related quality of life; however, no significant predictors were found in the long‐term (i.e., one‐year follow‐up) (Zonneveld et al. 2012). Negative affectivity, psychological attribution and anxiety were reduced by cognitive–behavioural, client‐centred or eclectic therapies (Kolk et al. 2004). Older age had a negative impact on physical functioning under MBCT if compared to EUC (Van Ravesteijn et al. 2013).

### 3.6 Somatization

Maroti et al. (2022) investigated patients with a somatization disorder (Maroti et al. 2022). The average age was 43 with females constituting 82% of the samples. The study took place in Sweden. The interventions were completed by 37 participants, who participated in 10 individual sessions lasting 100 min each of an internet‐based emotional awareness and expression therapy versus waiting list (Maroti et al. 2022).

#### 3.6.1 Predicting Primary Outcomes

Pain intensity was reduced as a result of the internet based emotional awareness and expression therapy compared to waiting list, with a mediation effect of depressive symptoms (Maroti et al. 2022).

### 3.7 Dissociative Seizures

One hundred and eighty patients with dissociative seizures, diagnosed after video‐electroencephalogram, were studied in Great Britain. Most of the sample (72%) was female, the average age was 37 years. The study compared the effects of 12 individual CBT sessions lasting 60 min each and combined with clinical management versus clinical management alone (Goldstein et al. 2022).

#### 3.7.1 Predicting Primary Outcomes

None of the baseline variables, including symptom severity, mental comorbidity or sex, significantly predicted dissociative seizure frequency at 12 months (Goldstein et al. 2022).

#### 3.7.2 Predicting Secondary Outcomes

Improvements in mental health‐related quality of life were higher in those with lower baseline depression or anxiety, who held a strong belief in the diagnosis and higher expectations from CBT (Goldstein et al. 2022). For physical health‐related quality of life, greater improvements were seen in participants with a shorter duration of dissociative seizures and in those who had fewer symptoms (Goldstein et al. 2022). A reduction in work and social adjustment was observed under CBT plus clinical management compared to clinical management alone when participants were younger at onset of dissociative seizures, employed, or in education, and not receiving state disability benefits (Goldstein et al. 2022). For physical health‐related quality of life, greater improvements were seen in participants who were younger at onset of the disorder (Goldstein et al. 2022). Higher‐level of education lead to better results in both mental and physical health‐related quality of life (Goldstein et al. 2022).

### 3.8 Functional Dyspepsia

In Norway, 50 patients with functional dyspepsia were assigned to 10 individual sessions of cognitive therapy lasting 50 min each, compared to a control group who did not receive a specific therapy. The average age of participants was 40 years and 59% was female. Patients were diagnosed after endoscopy (Haug et al. 1994).

#### 3.8.1 Predicting Primary Outcomes

Duration and severity of symptoms of functional dyspepsia, psychological distress, psychiatric comorbidity, sex, age, education, were no significant predictors for a reduction of symptom severity (Haug et al. 1994).

## 4 Discussion

This systematic review displays a complex trajectory of predictors, which influence psychotherapy success of FD.

Most studies applied a CBT‐based intervention, mainly provided in a face‐to‐face setting while five research used an internet‐based format (Ljótsson et al. 2011; Hedman et al. 2013; Friesen et al. 2017; Axelsson, Andersson, et al. 2020; Maroti et al. 2022). Nineteen studies employed a traditional CBT approach (Haug et al. 1994; Prins et al. 2001; Kolk et al. 2004; Blanchard et al. 2006; Heins et al. 2010; Lackner et al. 2010; Tummers et al. 2010; Ljótsson et al. 2011; Nakao et al. 2011; Zonneveld et al. 2012; Goedendorp et al. 2013; Hedman et al. 2013; Karlsson et al. 2015; Vallejo et al. 2015; Friesen et al. 2017; Axelsson, Hesser, et al. 2020; Goldstein et al. 2022), three used an emotional based approach (Weck et al. 2015; Richtberg et al. 2017; Maroti et al. 2022) and three studies adopted mindfulness‐based cognitive therapy (Van Ravesteijn et al. 2013; Andrés‐Rodríguez et al. 2019; Henrich et al. 2020). One study applied operant behavioural therapy (Thieme et al. 2007). Only in one case a psychodynamic therapy was used (Guthrie et al. 1991). The large representation of studies using CBT‐based interventions mirrors the wide evidence of clinical benefits of CBT for FD. The research field warrants further development because different variants and ingredients of CBT can be implemented and are in need of being tested for clinical utility, and because the literature on approaches other than CBT is almost lacking.

Four studies applied a three‐arm design, comparing two psychological interventions against waiting list or TAU (Prins et al. 2001; Vallejo et al. 2015; Weck et al. 2015; Andrés‐Rodríguez et al. 2019). Most studies had two arms comparing the experimental psychological intervention with waiting list (Guthrie et al. 1991; Ljótsson et al. 2011; Zonneveld et al. 2012; Goedendorp et al. 2013; Karlsson et al. 2015; Friesen et al. 2017; Axelsson, Hesser, et al. 2020; Henrich et al. 2020; Maroti et al. 2022) or TAU (Haug et al. 1994; Kolk et al. 2004; Tummers et al. 2010; Nakao et al. 2011; Goldstein et al. 2022). One study applied enhanced care (Van Ravesteijn et al. 2013) and another study used attention placebo (Thieme et al. 2007). All studies had a broad number of therapeutic sessions also varying in terms of time duration per session. Such heterogeneity shows that several variants of CBT‐based approach delivered via varying numbers of sessions can be effective, thus increasing the probability to provide to patients in real life an effective intervention besides the different availability of time of clinicians operating in the health system.

In some cases, no difference in predictors of treatment success was found between the experimental condition and the comparator, as shown for a CBT‐based intervention compared to TAU in CFS (Tummers et al. 2010), when CBT was compared to ET or to waiting list in hypochondriasis (Weck et al. 2015; Richtberg et al. 2017), or for EUC vs. TAU in MUS (Kolk et al. 2004). This underscores the need for further research to establish the efficacy of specific psychotherapeutic interventions for distinct FD diagnoses and to define which approaches allow for the reliable identification of treatment success predictors. It also underscores the need for a nuanced understanding of treatment efficacy, which includes, among the others, a detailed knowledge of individual patient characteristics such as baseline symptom severity, comorbidities, illness perceptions and treatment expectations. Considering these individual factors may help refine treatment allocation strategies and improve therapeutic outcomes in FD management.

Predictors of treatment success spanned various themes: sociodemographic variables, mental comorbidities, symptoms number/intensity, physical condition/functional impairments, emotional/cognitive/behavioural aspects, treatment‐related factors and FD diagnosis. Besides this wide range, pain intensity is a common predictor for treatment success of both FM and somatization. Depression and anxiety disorders were predictors for FM, IBS, somatization disorder, hypochondriasis, MUS and dissociative seizures. Overall, higher pre‐treatment symptom severity predicted worse outcomes (Heins et al. 2010; Lackner et al. 2010; Nakao et al. 2011; Friesen et al. 2017). This is in line with the recently published review by Löwe et al. (2024) showing that longer symptom duration, higher baseline symptom severity and lower physical, emotional or social functioning prior to treatment are associated with a poor treatment outcome. Further on, psychological predictors such as comorbid depressive or anxiety disorders, heightened symptom focus, catastrophizing interpretations, excessive worrying, somatosensory amplification and low symptom acceptance or self‐efficacy are associated with worse outcomes. Additionally, social determinants such as low educational level, lower socioeconomic status and limited access to adequate healthcare also contribute to unfavourable treatment outcomes (Löwe et al. 2024). Such a difference of predictors mirrors the need of keeping a broad view, which follows the lessons of the biopsychosocial model (Engel 1977) and which is more representative of complexity of real world clinical care paths. However, together with lack of standardized research methodology, it prevented meta‐analysing the present data.

Literature encompassing global research as well as studies outside the FD spectrum confirms that predictors of treatment outcomes in mental health results in a considerable heterogeneity in predictors of treatment outcomes, emphasizing that this variability is not unique to FD (Tanguay‐Sela et al. 2022; McDonald et al. 2023).

Initial concepts to address challenges in identifying predictors are emerging. However, additional knowledge may derive by strategies already proposed for mental disorders, such as multivariable predictive models using randomized trials (Chowdhury and Turin 2020) or machine learning (Lee et al. 2018; Sajjadian et al. 2021), and could be more systematically used in research on FD (Melidis et al. 2018). In addition, collaboration among researchers seem necessary to overcome barriers in establishing a common clinical and research background (e.g., EURONET‐SOMA—(Kohlmann et al. 2018) ETUDE—(Rosmalen et al. 2021a; Rosmalen et al. 2021b).

The present study has limitations. The exclusive consideration of randomized controlled trials for eligibility may have led to a limited inclusion of research. However, this choice ensured to use reliable findings being the product of the most rigorous design to verify efficacy of therapies. The restriction to psychotherapy might have narrowed the understanding of predictors of treatments. However, this choice was guided by the intention to select a rather homogeneous group of structured psychological therapies.

## 5 Conclusion and Future Directions

This systematic review underscores the complexity of predictors influencing psychotherapy success in patients with FD, identifying predictors for the successful treatment, that is pain intensity, mental comorbidity and symptom severity. These predictors can guide clinicians' treatment choices towards successful approaches as well as personalized, patient‐centred care paths; however, the heterogeneity of methods and findings across studies highlight the need for further research. Future studies might aim to refine predictive models using standardized methodologies, multivariable analyses, and machine learning approaches. Additionally, the present research highlights the need for further exploration preferably taking the advantage of a common vision among international and interdisciplinary scientists and clinicians. By integrating these insights, patient‐centred care and treatment outcomes can be optimized for individuals with FD.

## Author Contributions

Rometsch, Martin, Cosci: conceptualization, methodology. Rometsch: writing – original draft. Cosci, Martin: supervision, reviewing and editing.

## Ethics Statement

Ethical approval was not required for this study as it is a systematic review of the literature and did not involve the collection of new data from human participants or animals.

## Consent

Patient consent was not required for this study as it is a systematic review of the literature and did not involve the collection of data from individual patients.

## Permission to Reproduce Material From Other Sources

This systematic review does not include copyrighted material owned by third parties requiring reproduction permissions. All included materials have been appropriately cited, and no copyrighted excerpts necessitating permission for reproduction are presented in this manuscript.

## Supporting information


**Table S1:** Included studies with reports on predictors of treatment success.
**Table S2:** PICOS criteria for included studies on predictors of treatment success.
**Table S3:** Search terms for PubMed with subsequent filters:adults > 19 years.

## Data Availability

The data that support the findings of this study are available on request from the corresponding author. The data will also be made publicly available on the Open Science Framework (OSF) repository upon publication.

## References

[cpp70075-bib-0001] Abbass, A. , M. A. Lumley , J. Town , et al. 2021. “Short‐Term Psychodynamic Psychotherapy for Functional Somatic Disorders: A Systematic Review and Meta‐Analysis of Within‐Treatment Effects.” Journal of Psychosomatic Research 145: 110473. 33814192 10.1016/j.jpsychores.2021.110473

[cpp70075-bib-0002] Andrés‐Rodríguez, L. , X. Borràs , A. Feliu‐Soler , et al. 2019. “Immune‐Inflammatory Pathways and Clinical Changes in Fibromyalgia Patients Treated With Mindfulness‐Based Stress Reduction (MBSR): A Randomized, Controlled Clinical Trial.” Brain, Behavior, and Immunity 80: 109–119. 30818032 10.1016/j.bbi.2019.02.030

[cpp70075-bib-0003] Association, A. P . 2013. Diagnostic and Statistical Manual of Mental Disorders: DSM‐IV. American psychiatric association Washington.

[cpp70075-bib-0004] Axelsson, E. , E. Andersson , B. Ljótsson , D. Björkander , M. Hedman‐Lagerlöf , and E. Hedman‐Lagerlöf . 2020. “Effect of Internet vs. Face‐to‐Face Cognitive Behavior Therapy for Health Anxiety: A Randomized Noninferiority Clinical Trial.” JAMA Psychiatry 77, no. 9: 915–924. 32401286 10.1001/jamapsychiatry.2020.0940 PMC7221860

[cpp70075-bib-0005] Axelsson, E. , H. Hesser , E. Andersson , B. Ljótsson , and E. Hedman‐Lagerlöf . 2020. “Mediators of Treatment Effect in Minimal‐Contact Cognitive Behaviour Therapy for Severe Health Anxiety: A Theory‐Driven Analysis Based on a Randomised Controlled Trial.” Journal of Anxiety Disorders 69: 102172. 31864217 10.1016/j.janxdis.2019.102172

[cpp70075-bib-0006] Blanchard, E. B. , J. M. Lackner , R. Gusmano , et al. 2006. “Prediction of Treatment Outcome Among Patients With Irritable Bowel Syndrome Treated With Group Cognitive Therapy.” Behaviour Research and Therapy 44, no. 3: 317–337. 16413495 10.1016/j.brat.2005.01.003

[cpp70075-bib-0007] Burton, C. , P. Fink , P. Henningsen , B. Löwe , and W. Rief . 2020. “Functional Somatic Disorders: Discussion Paper for a New Common Classification for Research and Clinical Use.” BMC Medicine 18, no. 1: 1–7. 32122350 10.1186/s12916-020-1505-4 PMC7052963

[cpp70075-bib-0008] Carle‐Toulemonde, G. , J. Goutte , N. Do‐Quang‐Cantagrel , S. Mouchabac , C. Joly , and B. Garcin . 2023. “Overall Comorbidities in Functional Neurological Disorder: A Narrative Review.” L'encephale. 49: S24–S32. 10.1016/j.encep.2023.06.004 37414721

[cpp70075-bib-0010] Chaabouni, A. , J. Houwen , I. Walraven , et al. 2023. “Patients' Characteristics and General Practitioners' Management of Patients With Symptom Diagnoses.” Journal of the American Board of Family Medicine 36, no. 3: 477–492. 37290830 10.3122/jabfm.2022.220335R1

[cpp70075-bib-0011] Chowdhury, M. Z. I. , and T. C. Turin . 2020. “Variable Selection Strategies and Its Importance in Clinical Prediction Modelling.” Family Medicine and Community Health 8, no. 1: e000262. 32148735 10.1136/fmch-2019-000262 PMC7032893

[cpp70075-bib-0012] Dale, R. , K. Limburg , G. Schmid‐Mühlbauer , T. Probst , and C. Lahmann . 2023. “Somatic Symptom Distress and Gender Moderate the Effect of Integrative Group Psychotherapy for Functional Vertigo on Vertigo Handicap: A Moderation Analysis of a Randomised Controlled Trial.” Journal of Psychosomatic Research 167: 111175. 36753945 10.1016/j.jpsychores.2023.111175

[cpp70075-bib-0013] De Waal, M. W. M. , I. A. Arnold , J. A. Eekhof , and A. M. Van Hemert . 2004. “Somatoform Disorders in General Practice: Prevalence, Functional Impairment and Comorbidity With Anxiety and Depressive Disorders.” British Journal of Psychiatry 184, no. 6: 470–476. 10.1192/bjp.184.6.470 15172939

[cpp70075-bib-0014] Drossman, D. A. , and W. G. Thompson . 1992. “The Irritable Bowel Syndrome: Review and a Graduated Multicomponent Treatment Approach.” Annals of Internal Medicine 116, no. 12_Part_1: 1009–1016. 1586090 10.7326/0003-4819-116-12-1009

[cpp70075-bib-0015] Engel, G. L. 1977. “The Need for a new Medical Model: A Challenge for Biomedicine.” Science 196, no. 4286: 129–136. 847460 10.1126/science.847460

[cpp70075-bib-0016] Fava, G. A. , F. Cosci , and N. Sonino . 2017. “Current Psychosomatic Practice.” Psychotherapy and Psychosomatics 86, no. 1: 13–30. 27884006 10.1159/000448856

[cpp70075-bib-0017] Fava, G. A. , H. J. Freyberger , P. Bech , et al. 1995. “Diagnostic Criteria for Use in Psychosomatic Research.” Psychotherapy and Psychosomatics 63: 1–8. 7740096 10.1159/000288931

[cpp70075-bib-0018] Flückiger, C. , B. E. Wampold , J. Delgadillo , J. Rubel , A. Vîslă , and W. Lutz . 2020. “Is There an Evidence‐Based Number of Sessions in Outpatient Psychotherapy?—A Comparison of Naturalistic Conditions Across Countries.” Psychotherapy and Psychosomatics 89, no. 5: 333–335. 32403101 10.1159/000507793 PMC7490483

[cpp70075-bib-0065] Ford, A. C. , E. M. Quigley , B. E. Lacy , et al. 2014. “Effect of Antidepressants and Psychological Therapies, Including Hypnotherapy, in Irritable Bowel Syndrome: Systematic Review and Meta‐Analysis.” American Journal of Gastroenterology 109, no. 9: 1350–1365. 24935275 10.1038/ajg.2014.148

[cpp70075-bib-0019] Friedman, L. M. , C. D. Furberg , D. L. DeMets , D. M. Reboussin , and C. B. Granger . 2015. Fundamentals of Clinical Trials. Cham: Springer.

[cpp70075-bib-0020] Friesen, L. N. , H. D. Hadjistavropoulos , L. H. Schneider , N. M. Alberts , N. Titov , and B. F. Dear . 2017. “Examination of an Internet‐Delivered Cognitive Behavioural Pain Management Course for Adults With Fibromyalgia: A Randomized Controlled Trial.” Pain 158, no. 4: 593–604. 27984490 10.1097/j.pain.0000000000000802

[cpp70075-bib-0021] Gergov, V. , N. Lindberg , J. Lahti , J. Lipsanen , and M. Marttunen . 2021. “Effectiveness and Predictors of Outcome for Psychotherapeutic Interventions in Clinical Settings Among Adolescents.” Frontiers in Psychology 12: 628977. 33664698 10.3389/fpsyg.2021.628977 PMC7921706

[cpp70075-bib-0022] Goedendorp, M. M. , S. P. van der Werf , G. Bleijenberg , M. Tummers , and H. Knoop . 2013. “Does Neuropsychological Test Performance Predict Outcome of Cognitive Behavior Therapy for Chronic Fatigue Syndrome and What Is the Role of Underperformance?” Journal of Psychosomatic Research 75, no. 3: 242–248. 23972413 10.1016/j.jpsychores.2013.07.011

[cpp70075-bib-0023] Goldstein, L. , E. Robinson , T. Chalder , et al. 2022. “Moderators of Cognitive Behavioural Therapy Treatment Effects and Predictors of Outcome in the CODES Randomised Controlled Trial for Adults With Dissociative Seizures.” Journal of Psychosomatic Research 158: 110921. 35617911 10.1016/j.jpsychores.2022.110921

[cpp70075-bib-0024] Guthrie, E. , F. Creed , D. Dawson , and B. Tomenson . 1991. “A Controlled Trial of Psychological Treatment for the Irritable Bowel Syndrome.” Gastroenterology 100, no. 2: 450–457. 1985041 10.1016/0016-5085(91)90215-7

[cpp70075-bib-0025] Haug, T. T. , I. Wilhelmsen , S. Svebak , A. Berstad , and H. Ursin . 1994. “Psychotherapy in Functional Dyspepsia.” Journal of Psychosomatic Research 38, no. 7: 735–744. 7877128 10.1016/0022-3999(94)90026-4

[cpp70075-bib-0026] Häuser, W. , K. Bernardy , B. Arnold , M. Offenbächer , and M. Schiltenwolf . 2009. “Efficacy of Multicomponent Treatment in Fibromyalgia Syndrome: A Meta‐Analysis of Randomized Controlled Clinical Trials.” Arthritis Care & Research 61, no. 2: 216–224. 19177530 10.1002/art.24276

[cpp70075-bib-0027] Hedman, E. , N. Lindefors , G. Andersson , et al. 2013. “Predictors of Outcome in Internet‐Based Cognitive Behavior Therapy for Severe Health Anxiety.” Behaviour Research and Therapy 51, no. 10: 711–717. 23994252 10.1016/j.brat.2013.07.009

[cpp70075-bib-0028] Heins, M. J. , H. Knoop , J. B. Prins , M. Stulemeijer , J. W. Van Der Meer , and G. Bleijenberg . 2010. “Possible Detrimental Effects of Cognitive Behaviour Therapy for Chronic Fatigue Syndrome.” Psychotherapy and Psychosomatics 79, no. 4: 249–256. 20502065 10.1159/000315130

[cpp70075-bib-0029] Henrich, J. F. , B. Gjelsvik , C. Surawy , E. Evans , and M. Martin . 2020. “A Randomized Clinical Trial of Mindfulness‐Based Cognitive Therapy for Women With Irritable Bowel Syndrome—Effects and Mechanisms.” Journal of Consulting and Clinical Psychology 88, no. 4: 295–310. 32134291 10.1037/ccp0000483

[cpp70075-bib-0030] Karlsson, B. , G. Burell , U.‐M. Anderberg , and K. Svärdsudd . 2015. “Cognitive Behaviour Therapy in Women With Fibromyalgia: A Randomized Clinical Trial.” Scandinavian Journal of Pain 9, no. 1: 11–21. 29911653 10.1016/j.sjpain.2015.04.027

[cpp70075-bib-0031] Kleinstaeuber, M. , M. Witthoeft , A. Steffanowski , H. van Marwijk , W. Hiller , and M. J. Lambert . 2014. “Pharmacological Interventions for Somatoform Disorders in Adults.” Cochrane Database of Systematic Reviews 2014, no. 11: CD010628. 25379990 10.1002/14651858.CD010628.pub2 PMC11023023

[cpp70075-bib-0032] Kohlmann, S. , B. Löwe , and M. C. Shedden‐Mora . 2018. “Health Care for Persistent Somatic Symptoms Across Europe: A Qualitative Evaluation of the EURONET‐SOMA Expert Discussion.” Frontiers in Psychiatry 9: 646. 30581394 10.3389/fpsyt.2018.00646 PMC6292948

[cpp70075-bib-0033] Kolk, A. , S. Schagen , and G. Hanewald . 2004. “Multiple Medically Unexplained Physical Symptoms and Health Care Utilization: Outcome of Psychological Intervention and Patient‐Related Predictors of Change.” Journal of Psychosomatic Research 57, no. 4: 379–389. 15518674 10.1016/j.jpsychores.2004.02.012

[cpp70075-bib-0034] Kustra‐Mulder, A. , B. Löwe , and A. Weigel . 2023. “Healthcare‐Related Factors Influencing Symptom Persistence, Deterioration, or Improvement in Patients With Persistent Somatic Symptoms: A Scoping Review of European Studies.” Journal of Psychosomatic Research 111485. 10.1016/j.jpsychores.2023.111485 37716128

[cpp70075-bib-0035] Lackner, J. M. , G. D. Gudleski , L. Keefer , S. S. Krasner , C. Powell , and L. A. Katz . 2010. “Rapid Response to Cognitive Behavior Therapy Predicts Treatment Outcome in Patients With Irritable Bowel Syndrome.” Clinical Gastroenterology and Hepatology 8, no. 5: 426–432. 20170751 10.1016/j.cgh.2010.02.007 PMC3144205

[cpp70075-bib-0036] Lagrand, T. J. , M. Jones , A. Bernard , and A. C. Lehn . 2023. “Health Care Utilization in Functional Neurologic Disorders: Impact of Explaining the Diagnosis of Functionals Seizures on Health Care Costs.” Neurology: Clinical Practice 13, no. 1: e200111. 36865642 10.1212/CPJ.0000000000200111 PMC9973286

[cpp70075-bib-0037] Lee, Y. , R.‐M. Ragguett , R. B. Mansur , et al. 2018. “Applications of Machine Learning Algorithms to Predict Therapeutic Outcomes in Depression: A Meta‐Analysis and Systematic Review.” Journal of Affective Disorders 241: 519–532. 30153635 10.1016/j.jad.2018.08.073

[cpp70075-bib-0038] Liu, J. , N. S. Gill , A. Teodorczuk , Z.‐J. Li , and J. Sun . 2019. “The Efficacy of Cognitive Behavioural Therapy in Somatoform Disorders and Medically Unexplained Physical Symptoms: A Meta‐Analysis of Randomized Controlled Trials.” Journal of Affective Disorders 245: 98–112. 30368076 10.1016/j.jad.2018.10.114

[cpp70075-bib-0039] Ljótsson, B. , G. Andersson , E. Andersson , et al. 2011. “Acceptability, Effectiveness, and Cost‐Effectiveness of Internet‐Based Exposure Treatment for Irritable Bowel Syndrome in a Clinical Sample: A Randomized Controlled Trial.” BMC Gastroenterology 11, no. 1: 1–13. 21992655 10.1186/1471-230X-11-110 PMC3206465

[cpp70075-bib-0040] Löwe, B. , A. Toussaint , J. G. Rosmalen , et al. 2024. “Persistent Physical Symptoms: Definition, Genesis, and Management.” Lancet 403, no. 10444: 2649–2662. 38879263 10.1016/S0140-6736(24)00623-8

[cpp70075-bib-0041] Mamo, N. , M. van de Klundert , L. Tak , T. O. Hartman , D. Hanssen , and J. Rosmalen . 2023. “Characteristics of Collaborative Care Networks in Functional Disorders: A Systematic Review.” Journal of Psychosomatic Research 111357. 10.1016/j.jpsychores.2023.111357 37392482

[cpp70075-bib-0042] Maroti, D. , M. A. Lumley , H. Schubiner , et al. 2022. “Internet‐Based Emotional Awareness and Expression Therapy for Somatic Symptom Disorder: A Randomized Controlled Trial.” Journal of Psychosomatic Research 163: 111068. 36327532 10.1016/j.jpsychores.2022.111068

[cpp70075-bib-0043] McDonald, S. , M. Melkonian , E. Karin , B. F. Dear , N. Titov , and B. M. Wootton . 2023. “Predictors of Response to Cognitive Behavioural Therapy (CBT) for Individuals With Obsessive‐Compulsive Disorder (OCD): A Systematic Review.” Behavioural and Cognitive Psychotherapy: 1–18. 10.1017/S1352465823000103 37013903

[cpp70075-bib-0044] Melidis, C. , S. L. Denham , and M. E. Hyland . 2018. “A Test of the Adaptive Network Explanation of Functional Disorders Using a Machine Learning Analysis of Symptoms.” Biosystems 165: 22–30. 29278731 10.1016/j.biosystems.2017.12.010

[cpp70075-bib-0045] Methley, A. M. , S. Campbell , C. Chew‐Graham , R. McNally , and S. Cheraghi‐Sohi . 2014. “PICO, PICOS and SPIDER: A Comparison Study of Specificity and Sensitivity in Three Search Tools for Qualitative Systematic Reviews.” BMC Health Services Research 14, no. 1: 1–10. 25413154 10.1186/s12913-014-0579-0 PMC4310146

[cpp70075-bib-0046] Nakao, M. , Y. Shinozaki , D. K. Ahern , and A. J. Barsky . 2011. “Anxiety as a Predictor of Improvements in Somatic Symptoms and Health Anxiety Associated With Cognitive‐Behavioral Intervention in Hypochondriasis.” Psychotherapy and Psychosomatics 80, no. 3: 151–158. 21372623 10.1159/000320122

[cpp70075-bib-0047] Ouzzani, M. , H. Hammady , Z. Fedorowicz , and A. Elmagarmid . 2016. “Rayyan—A Web and Mobile App for Systematic Reviews.” Systematic Reviews 5, no. 1: 1–10. 27919275 10.1186/s13643-016-0384-4 PMC5139140

[cpp70075-bib-0048] Page, M. J. , J. E. McKenzie , P. M. Bossuyt , et al. 2021. “The PRISMA 2020 Statement: An Updated Guideline for Reporting Systematic Reviews.” International Journal of Surgery 88: 105906. 33789826 10.1016/j.ijsu.2021.105906

[cpp70075-bib-0049] Prins, J. B. , G. Bleijenberg , E. Bazelmans , et al. 2001. “Cognitive Behaviour Therapy for Chronic Fatigue Syndrome: A Multicentre Randomised Controlled Trial.” Lancet 357, no. 9259: 841–847. 11265953 10.1016/S0140-6736(00)04198-2

[cpp70075-bib-0050] Richtberg, S. , M. Jakob , V. Höfling , and F. Weck . 2017. “Patient Characteristics and Patient Behavior as Predictors of Outcome in Cognitive Therapy and Exposure Therapy for Hypochondriasis.” Journal of Clinical Psychology 73, no. 6: 612–625. 27532367 10.1002/jclp.22356

[cpp70075-bib-0063] Rometsch, C. , G. Mansueto , F. Maas Genannt Bermpohl , A. Martin , and F. Cosci . 2024. “Prevalence of Functional Disorders Across Europe: A Systematic Review and Meta‐Analysis.” European Journal of Epidemiology 39, no. 6: 571–586. 38551715 10.1007/s10654-024-01109-5 PMC11249491

[cpp70075-bib-0051] Rosmalen, J. , C. Burton , A. Carson , et al. 2021a. “The European Training Network ETUDE (Encompassing Training in Functional Disorders Across Europe) Is Recruiting 15 Early‐Stage Researchers.” Psychotherapy and Psychosomatics 90, no. 2: 142–144. 10.1016/j.jpsychores.2020.110345 33385705

[cpp70075-bib-0052] Rosmalen, J. , C. Burton , A. Carson , et al. 2021b. “The European Training Network ETUDE (Encompassing Training in fUnctional Disorders across Europe): A New Research and Training Program of the EURONET‐SOMA Network Recruiting 15 Early Stage Researchers.” Journal of Psychosomatic Research 141: 110345. 33385705 10.1016/j.jpsychores.2020.110345

[cpp70075-bib-0053] Sajjadian, M. , R. W. Lam , R. Milev , et al. 2021. “Machine Learning in the Prediction of Depression Treatment Outcomes: A Systematic Review and Meta‐Analysis.” Psychological Medicine 51, no. 16: 2742–2751. 35575607 10.1017/S0033291721003871

[cpp70075-bib-0054] Sarter, L. , J. Heider , M. Witthöft , W. Rief , and M. Kleinstäuber . 2022. “Using Clinical Patient Characteristics to Predict Treatment Outcome of Cognitive Behavior Therapies for Individuals With Medically Unexplained Symptoms: A Systematic Review and Meta‐Analysis.” General Hospital Psychiatry 77: 11–20. 35390568 10.1016/j.genhosppsych.2022.03.001

[cpp70075-bib-0055] Saunders, C. , H. Treufeldt , M. T. Rask , et al. 2023. “Explanations for Functional Somatic Symptoms Across European Treatment Settings: A Mixed Methods Study.” Journal of Psychosomatic Research 166: 111155. 36680846 10.1016/j.jpsychores.2023.111155

[cpp70075-bib-0056] Tanguay‐Sela, M. , C. Rollins , T. Perez , et al. 2022. “A Systematic meta‐Review of Patient‐Level Predictors of Psychological Therapy Outcome in Major Depressive Disorder.” Journal of Affective Disorders 317: 307–318. 36029877 10.1016/j.jad.2022.08.041

[cpp70075-bib-0057] Thieme, K. , D. C. Turk , and H. Flor . 2007. “Responder Criteria for Operant and Cognitive–Behavioral Treatment of Fibromyalgia Syndrome.” Arthritis Care & Research: Official Journal of the American College of Rheumatology 57, no. 5: 830–836. 10.1002/art.22778 17530683

[cpp70075-bib-0058] Tummers, M. , H. Knoop , and G. Bleijenberg . 2010. “Effectiveness of Stepped Care for Chronic Fatigue Syndrome: A Randomized Noninferiority Trial.” Journal of Consulting and Clinical Psychology 78, no. 5: 724–731. 20873907 10.1037/a0020052

[cpp70075-bib-0059] Vallejo, M. A. , J. Ortega , J. Rivera , M. I. Comeche , and L. Vallejo‐Slocker . 2015. “Internet Versus Face‐To‐Face Group Cognitive‐Behavioral Therapy for Fibromyalgia: A Randomized Control Trial.” Journal of Psychiatric Research 68: 106–113. 26228408 10.1016/j.jpsychires.2015.06.006

[cpp70075-bib-0060] Van Ravesteijn, H. , P. Lucassen , H. Bor , C. Van Weel , and A. Speckens . 2013. “Mindfulness‐Based Cognitive Therapy for Patients With Medically Unexplained Symptoms: A Randomized Controlled Trial.” Psychotherapy and Psychosomatics 82, no. 5: 299–310. 23942259 10.1159/000348588

[cpp70075-bib-0061] Weck, F. , J. M. Neng , J. Schwind , and V. Höfling . 2015. “Exposure Therapy Changes Dysfunctional Evaluations of Somatic Symptoms in Patients With Hypochondriasis (Health Anxiety). A Randomized Controlled Trial.” Journal of Anxiety Disorders 34: 1–7. 26093823 10.1016/j.janxdis.2015.05.008

[cpp70075-bib-0064] World Health Organization . 1992. The ICD‐10 Classification of Mental and Behavioural Disorders: Clinical Descriptions and Diagnostic Guidelines. World Health Organization.

[cpp70075-bib-0062] Zonneveld, L. N. , Y. R. van Rood , C. G. Kooiman , R. Timman , A. van't Spijker , and J. J. Busschbach . 2012. “Predicting the Outcome of a Cognitive‐Behavioral Group Training for Patients With Unexplained Physical Symptoms: A One‐Year Follow‐Up Study.” BMC Public Health 12: 1–10. 23039913 10.1186/1471-2458-12-848 PMC3549894

