# Supplementary material for: Predictors of Treatment Success of Psychotherapy in Functional Disorders: A Systematic Review of the Literature
Source: Clin Psychol Psychother. 2025 Apr 23;32(2):e70075. doi: 10.1002/cpp.70075 (PMC12018217; doi:10.1002/cpp.70075)
Supplement: Supplementary file 1 — Table S1: Included studies with reports on predictors of treatment success. Table S2: PICOS criteria for included studies on predictors of treatment success. Table S3: Search terms for PubMed with subsequent filters:adults > 19 years. [file CPP-32-e70075-s001.docx]

# Predictors of treatment success of psychological interventions in functional disorders: A systematic review of the literature

Short running head: Predictors of treatment success in functional disorders

Caroline Rometsch^1­^, MD, M. Sc., Alexandra Martin^2^, PhD, Fiammetta Cosci^3,4^, MD, PhD

^1^ Department of Experimental and Clinical Medicine, University of Florence, Italy

^2^ School of Human and Social Sciences, University of Wuppertal, Wuppertal, Germany

^3^ Department of Health Sciences, University of Florence, Italy

^4^ Department of Psychiatry and Neuropsychology, Maastricht University, Netherlands

**Corresponding author:**

Caroline Rometsch

Department of Experimental and Clinical Medicine

University of Florence

Largo Brambilla, 3

50134 Firenze

Italy

Carolina.Rometsch@unifi.it

ORCID:

Caroline Rometsch: 0000-0002-3172-0823

Fiammetta Cosci: 0000-0002-5022-0488

Alexandra Martin: 0000-0002-4235-8591

**Keywords:**

Functional disorders, predictor, treatment success, outcome, systematic review

# Online supplementary material

## S1: Included studies with reports on predictors of treatment success

| **Author** | **Diagnosis** | **Sample size** | **RCT details** | **Number of sessions (duration in minutes)** | **Statistical procedure** | **Statistical outcome** | **Dependent variables** | **Predictor variable** | **Results** | **Secondary results** |
| --- | --- | --- | --- | --- | --- | --- | --- | --- | --- | --- |
| Heins et al. (2010) | Chronic Fatigue Syndrome (CFS) based on the U.S. Center for Disease Control (CDC) | 508 | CBT vs. care-as-usual, support-groups, waiting-list | 12 weekly sessions of 60-90 | Chi-square test, independent two-sided t-tests, logistic regression model | R^2^ | Checklist Individual Strength, (CIS subscale), pain, Sickness Impact Profile, (SIP), Short Form 36, (SF-36), Symptom Checklist-90 (SCL-90) | Checklist Individual Strength (CIS), Sickness Impact Profile (SIP), physical attributions, and physical activity (actometer) | Predictors of symptom deterioration were high functional impairment (SIP) and physical attributions. Lower baseline fatigue (CIS) predicted deterioration in controls. High daily observed fatigue (DOF) predicted deterioration in both groups. | Lower baseline fatigue predicted deterioration in control groups, but this effect was smaller in the CBT group |
| Goedendorp et al. (2013) | Chronic Fatigue Syndrome (CFS) based on the U.S. Center for Disease Control (CDC) | 171 | Guided self-instruction + CBT vs. waiting period followed by regular CBT | 14 CBT sessions (duration per session not specified), guided self-instruction: 16 weeks | ANOVA, regression analysis, t-tests, Fisher’s exact test | Interaction effects, main effects of underperformance on functional impairments (SIP total), and physical limitations (SF-36) | CIS-fatigue, SIP total, SF-36 physical functioning | Underperformance (ASTMT score < 84), neuropsychological test performance (simple RT, choice RT, SDMT) | Predictor for dropout and greater functional impairments was underperformance on neuropsychological tests | Underperformance related to worse neuropsychological test performance, no significant effect on reduction of fatigue or disabilities after CBT |
| Prins et al. (2001) | CFS based on US Centers for Disease Control and Prevention | 278 | CBT vs. guided support groups vs. control group | CBT: 16 sessions, each lasting 1 hour, over 8 months;  Support groups: 11 meetings, each lasting 1.5 hours, over 8 months | Stepwise multiple linear regression was used for predictor analysis. | R^2^ | Checklist Individual Strength (CIS), Sickness Impact Profile (SIP) | Self-Efficacy Scale, Actometer, Symptom Checklist-90 (SCL-90), Causal Attribution List | Predictors for fatigue severity were a greater sense of control, which improved outcomes with CBT, while a passive activity pattern and focusing on bodily symptoms hindered improvement. Predictors for functional impairment included CBT, which improved outcomes, but focusing on bodily symptoms reduced this benefit. | A secondary outcome was the improvement in psychological well-being and quality of life. The relevant predictor variables for these outcomes were the patient's sense of control and reduction in focusing on bodily symptoms, both of which were associated with better psychological well-being and higher quality of life. |
| Tummers et al. (2010) | CFS (U.S. Centers for Disease Control criteria for CFS according to Fukuda et al., 1994) | 171 | CBT (stepped care) vs. TAU | 14 (NA) | Two logistic regression analyses | Interaction terms, and Coefficient of Determination (R^2^) with  Regression coefficient (B) and 95%CI | Checklist Individual Strength (CIS)  Sickness Impact Profile  Short Form–36 (SF-36) | Checklist Individual Strength (CIS) Fatigue Severity and condition | No significant predictor was found for the differences in fatigue severity between the stepped care and care as usual groups  No significant predictor was identified for determining the proportion of patients with a clinically significant improvement of fatigue post guided self-instruction when compared with stepped care and care as usual | Comparison of two further conditions: (a)  only guided self-instruction and stepped care,  (b) only guided  self-instruction and TAU on CIS Fatigue Severity  Result: after self-instruction, significant lower proportion with improvement compared to stepped care and TAU |
| Andrés-Rodríguez et al. (2019) | FM (ACR-1990 criteria) | 70 | 3 arms: TAU, TAU + MBSR, and TAU + FibroQoL | 8 weekly sessions (120 min/session) + optional 6-hour meditation retreat | REML Linear mixed models | regression coefficient (B) | FIQR, HADS, PSS-10, PCS, PIPS, MISCI, FFMQ, IL-6, CXCL8, IL-10, hs-CRP | Baseline levels of cytokines (IL-6, CXCL8, IL-10) | MBSR improved FM severity, maintained IL-10 levels, and higher baseline CXCL8 predicted reduced treatment efficacy attenuated effects of MBSR on clinical outcomes | MBSR increased mindfulness (FFMQ), reduced depression (HADS-D), stress (PSS-10), catastrophizing (PCS), and cognitive impairment (MISCI) |
| Friesen et al. (2017) | FM (ACR-1990 criteria) | 60 | Internet-delivered cognitive behavioral pain management program (Pain Course) vs. WL | 5 sessions (8 weeks) | Generalized Estimation Equation (GEE) | Wald's χ² | FM severity, pain, depression, anxiety, Fibromyalgia Impact Questionnaire (FIQR) | Treatment group | Significant improvements in FM, depression, anxiety, and pain (FIQR, GAD-7, PHQ-9, BPI) | Improvements in fear of movement, pain-related avoidance |
| Karlsson et al. (2015) | FM (ACR-1990 criteria) | 48 | CBT vs. WL | 20 sessions (180 minutes) | General Linear Model (Repeated Measures ANOVA) | Interaction effect | Life control: West Haven-Yale Multidimensional Pain Inventory (MPI-1),  Affective distress: West Haven-Yale Multidimensional Pain Inventory (MPI-1),  Depression: Montgomery-Åsberg Depression Rating Scale (MADRS | Being in gainful work, waist/hip ratio, smoking habits, alcohol consumption, duration of generalized pain, number of tender points, menstrual status, important childhood experiences | Improvement in life control, affective distress, interference, support from spouses in the intervention group vs control group | Improvement in depression, vital exhaustion, and stress behavior; no significant improvement in pain severity, but increased awareness of pain |
| Thieme et al. (2007) | FM (ACR-1990 criteria) | 125 | CBT vs. operant–behavioral therapy (OBT) vs. attention placebo | 15 (120) | Multinominal logistic regression analyses | Coefficient of Determination (R^2^)  Regression Coefficient (B)  Standard Error (SE) | Pain intensity | Pain severity (MPI)  Physical functioning (FIQ)  Pain intensity (MPI)  Affective distress (MPI)  Coping and  catastrophizing (PRSS)  Behavioral variables (solicitous  spouse responses in the MPI, pain behaviors, number of  physician visits)  Duration of pain | Significant pain reduction was predicted by low physical impairment prior to treatment, but no significant predicting outcome for variables:  duration of pain, psychological factors, and behavioral factors | Pain intensity was associated with lower initial physical impairment; high physical impairment prior to treatment decreased the likelihood of clinically significant improvement of pain (around 0.5 times) |
| Vallejo et al. (2015) | FM (ACR-1990 criteria) | 60 | Internet-delivered CBT vs. face-to-face CBT vs. WL | 10 (120) | Multi-level modelling analysis:  Two levels were measured, time effects on level 1 and treatment effects on level 2, level 1 variables were used to predict level 2 variables | Maximum Likelihood Estimation  Level 1 Effects (Time Effects)  Level 2 Effects (Treatment Effects) | Fibromyalgia Impact Questionnaire (FIQ) | Fibromyalgia Impact Questionnaire (FIQ)  BDI  Chronic Pain Self-efficacy Scale (CPSS)  Chronic Pain Coping Inventory (CPCI) | WL: Significant predictor magnification  CBT: Significant predictors for improvement:  FIQ, psychological distress, BDI, catastrophizing, rumination, helplessness and the use of relaxation as a coping resource  iCBT: like CBT plus  improved self-efficacy of pain, coping with symptoms, global self-efficacy, and magnification,  but no significant results to use of relaxation |  |
| Axelsson et al. (2020) | Hypochondriasis (DSM-5) | 204 | CBT vs. WL | 12 weeks | Parallel process growth modeling, cross-lagged panel models | Growth in mediators, no temporal mediation found | Health Anxiety Inventory | Non-reactivity, health anxiety behaviors (Five-Facet Mindfulness Questionnaire (FFMQ-NR)), perceived competence (Perceived Competence Scale (PCS)), somatosensory amplification (Somatosensory Amplification Scale (SSAS)) | A predictor for improved health anxiety was increased non-reactivity and perceived competence. No predictors led to future reductions in health anxiety. | Health anxiety influenced subsequent change in mediators but not vice versa. CBT reduced SHA behaviors and increased non-reactivity and competence. |
| Nakao et al. (2012) | Hypochondriasis (DSM-IV-TR) | 182 | CBT vs. usual-care control group | 6 sessions (90 minutes each) | Multiple regression analysis | β coefficient | Hypochondriasis scores (Whiteley Index, Health Anxiety Inventory, Somatic Symptom Inventory) | Pretreatment anxiety levels | A predictor for hypochondriasis score reduction was high pretreatment anxiety. | Being married also predicted better treatment outcomes on the Whiteley Index and Somatic Symptom Inventory, after adjusting for baseline depression and demographic factors. |
| Richtberg et al. (2017) | Hypochondrias (DSM-IV) | 75 | CBT vs. ET | 12 (50) | Hierarchical multiple regression analysis | Interaction terms with regression coefficients (B) and standard errors (SE) | Hypochondriasis Yale-Brown Obsessive-Compulsive Scale (H-YBOCS), Postscore | Prescore of the H-YBOCS  Sociodemographic  variables (sex, age, and cohabitation)  Comorbid mental disorder (depression or anxiety)  General psychopathology (BDI-II, Beck Anxiety Inventory (BAI), PHQ-15)  Assessment Form of Patient Interpersonal Behavior (AFPIB) | Significant predictors for the postscore of H-YBOCS:  1. pre- H-YBOCS  2. AFPIB score  Not significant:  Sociodemographic variables, comorbid mental disorders, general psychopathology | Significant predictors for the follow-Up Scores of the H-YBOCS:  1. comorbid mental disorders  2. AFPIB  Not significant:  pre-H-YBOCS score, sociodemographic variables, psychopathology |
| Weck et al. (2015) | Hypochondrias  (DSM-IV) | 73 | CT vs. ET vs. WL | 12 (NA) | Mediation analysis | Direct and indirect effects, R^2­­­^_,_ 95%CI | Illness Attitude Scales (IAS) | Condition (active treatment [ET and CT] or WL)  Mediator Variables:  Symptoms and Outcome Scale (SOS CAT score)  Health Norms Sorting Task (HNST)  HNSTspecific score  HNSTunspecific score | HNSTspecific significantly mediated the relationship between the treatment condition and the IAS score | Significant correlations:  1. SOS-CAT with IAS health anxiety scale  2. HNSTunspecific with IAS health anxiety scale  3. HNSTspecific with IAS health anxiety scale  Mediation:  SOS-CAT and HNSTunspecific did NOT significantly mediate the relationship between the condition and the IAS |
| Blanchard et al. (2006) | IBS (Rome II criteria) | 129 | Group cognitive therapy vs. psychoeducational support | 10 weekly, 90 minutes | Univariate analysis,  Multivariate regression analysis,  Hierarchical and stepwise regression analysis |  | IBS-specific Quality of Life (QOL),  Global Severity Index (GSI) | Predictor variables: Demographics (age, race, education), GI symptoms (baseline pain, tenderness, diarrhea), psychiatric status (Axis I, anxiety, depression), cognitive measures (DAS, ATQ), QOL (SF-36, baseline QOL), stress-related measures (DSI, HS), psychological measures (BDI, STAI, PSWQ) | Modest prediction for GI symptom improvement (4-15% variance), stronger prediction for QOL and psychological distress (36-50% variance), higher baseline anxiety, depression, and stress predicted poorer outcomes in QOL and psychological distress, psychopathology (Axis I disorders) associated with less GI symptom improvement. | Greater improvement in QOL was related to less depression, better social functioning, and lower baseline diarrhea; Older age was a predictor of poorer bowel regularity improvement |
| Guthrie et al. (1991) | IBS (via gastroenterologist) | 102 |  | 7 (120) | Discriminant Function Analysis | predicted group membership and correlation coefficients | Bowel symptoms (i.e., severity abdominal distention, diarrhea)  Psychiatric symptoms: anxiety and depression | Pain: (constant or episodic)  Recognition of stress  Psychiatric diagnosis (anxiety/depression)  Duration of symptoms  Sites of abdominal pain | Significant predictors of improvement were:  Not describing pain as constant (i.e., having discernible episodes)  Recognizing that pain was exacerbated by stress  Diagnosis of anxiety and/or depression |  |
| Henrich et al. (2020) | IBS  (Rome III) | 67 | Mindfulness for IBS (MIBS) vs. WL | 6 (120) | Discriminant Function Analysis | Predicted Group Membership, Canonical Correlation | Visceral anxiety sensitivity (VSI)  Pain-catastrophizing (PCS)  Implicit Association Test (IAT-D, (changes from T1 to T2)  Nonjudgmental awareness scores (FFMQ-24, changes from T1 to T3) | Time and treatment group | VSI significantly mediated the effects of MIBS on IBS symptom severity  Not significant: PCS  Self-referential processing of illness/health and nonjudgmental awareness played roles in mediating the effect of MIBS on IBS symptom severity, but this mediation effect was not robust to controlling for baseline levels | Treatment expectations, but not credibility, were associated with improvements in symptoms and quality of life |
| Lackner et al. (2010) | IBS (Rome II) | 71 | CBT in two formats: 10 weekly sessions or 4 sessions over 10 weeks vs. WL | 10 weekly sessions or 4 sessions (60 minutes) | One-way ANOVAs, Chi-square, MANOVA | F-values and P-values | IBS symptom severity, Quality of Life (QOL) | Rapid response at week 4:  1 Reported adequate relief of pain and IBS symptoms.  2 Had a 50+ point reduction in IBS severity | Rapid responders (RRs) showed sustained improvement in IBS symptoms and QOL, maintained at 3-month follow-up | RRs had higher baseline symptom severity but showed better sustained response compared to non-rapid responders |
| Ljótsson et al. (2011) | IBS (Rome III) | 61 | Internet-based cognitive behavior therapy (ICBT) vs. WL | 5 (NA) | Linear Mixed Effects Model | Standardized Mean Differences (Cohen's d) | Symptom severity:  Improvement on the GSRS-IBS (Gastrointestinal Symptom Rating Scale for Irritable Bowel Syndrome) | Visceral Sensitivity Index (VSI)  Irritable Bowel Syndrome Quality of Life (IBS-QOL)  Sheehan Disability Scales (SDS) | No significant predictors: VSI, IBS-QOL, SDS for predicting improvement in GSRS-IBS scores in the ICBT group |  |
| Kolk et al. (2004) | MUS (self-defined criteria) | 98 | Psychological intervention + TAU vs. TAU | 12 (60) | Path analysis (using LISREL 8) | Standardized Path Coefficients | Change in self-reported unexplained symptoms  (Symptom Checklist 90 (SCL-90))  Change in registered unexplained symptoms  Change in GP consultations | Age  Gender  SES (Socioeconomic Status)  Chronic disease  Negative affectivity Positive and Negative Affectivity Schedule (PANAS)  Symptom Checklist 90 (SCL-90) Selective attention  Employment status  Housekeeping status  Psychological attribution Symptom Interpretation Questionnaire (SIQ)  Somatic attribution Symptom Interpretation Questionnaire (SIQ)  Anxiety | Significant Predictors:  Pretreatment anxiety: Direct effect on the decrease of self-reported unexplained symptoms  Somatic attribution: Less somatic attribution directly increased symptom reduction  Negative affectivity: Indirectly predicted symptom reduction via more psychological attribution and more pretreatment anxiety  Psychological attribution: had a positive relationship with anxiety  Registered symptoms: Had a direct effect on changes in consultations | Non-significant Predictors:  Age/Gender/SES/chronical diseases, employment status, housekeeping: not significant for symptom change  Selective attention: not significant for symptom reduction  Change in self-reported unexplained symptoms: not significant for change in registered symptoms and consultations |
| Van Ravesteijn et al. (2013) | MUS (DSM-IV) | 125 | Mindfulness-based cognitive therapy (MBCT) vs. ECU | 8 (150) | Predicting Factors Analysis with subgroup analysis | Interaction terms with 95%CI | Visual analogue scale (VAS),  EuroQol 5D (EQ-5D),  Mental component summary (MCS),  physical component summary | Group (MBCT vs. EUC)  Age  Gender  Level of education  Presence of physical diseases  Presence of additional psychiatric disorders | Significant Interaction:  Age had a significant interaction with physical functioning with increase in age being negatively associated with physical functioning in the MBCT group compared to the EUC group. | Non-significant Interactions:  Gender, level of education, the presence of physical diseases, and the presence of psychiatric disorders did not have significant interactions with the three main outcome measures (VAS EQ-5D, MCS, and physical component summary). |
| Zonneveld et al. (2012) | MUS (DSM-IV) | 162 | CBT vs. WL | Weekly 2-hour sessions over 13 weeks | Hierarchical multiple regression analyses | R² values | Physical Component Summary (PCS) of SF-36 (quality of life in the physical domain) | Psychological symptoms (SCL-90-R), personality-disorder characteristics (VKP), psychiatric history (SCID-I), health-related quality of life in the mental domain (MCS of SF-36) | Predictors were significant for short-term outcome (explained 15% of variance); better outcomes were associated with more psychological symptoms, fewer personality-disorder characteristics, the presence of a psychiatric history, and better mental health-related quality of life | No significant prediction for long-term outcomes |
| Maroti et al. (2022) | Somatic symptom disorder (DSM-5) | 74 | CBT (internet-based emotional awareness and expression therapy (I-EAET)) vs. WL | 10 (100) | Mediator analysis | Direct and indirect effects with 95%CI | I-EAET vs. WL | Somatic symptoms (PHQ-15) and pain intensity (BPI-4)  Mediators: Emotional processing (EPS-25),  Depression (PHQ-9) | Significant mediators:  1. Signs of unprocessed emotions (EPS-25)  2. Depressive symptoms (PHQ-9)  on the relation between group level and somatic symptom reduction | Total proportion of the explained effect of those two mediators: 72% |
| Goldstein et al. (2022) | Dissociative seizures (DS) (ICD-10) | 368 | CBT and standardized medical care (SMC) vs. SMC | 12 (60) | Mixed effects multiple linear regression and  interaction analysis | Interaction terms, Coefficient of Determination (R^2^),  regression coefficient (B) with incidence rate ratios | Monthly seizure frequency  Work and Social Adjustment Scale (WSAS)  Mental Component Summary from the Short Form 12 Health Survey (SF-12v2 MCS)  Physical Component Summary from the Short Form 12 Health Survey (SF-12v2 PCS) | Main Predictor: Trial arm  Moderators:  Gender, PHQ-15, M.I.N.I. diagnosis | Not significant: no baseline variables predicted DS frequency at 12 months  Significant predictors of improved outcome were:  A status of not receiving disability benefits (for PCS, MCS, and WSAS outcomes)  Lower scores in anxiety and/or depression (for PCS, MCS, and WSAS outcomes)    A shorter duration since the onset of DS (for PCS and WSAS outcomes)    A younger age at the time DS first manifested (for PCS and WSAS outcomes)  Being employed or actively in education (for PCS and WSAS outcomes)  A reduced number of symptoms as determined by the Modified PHQ-15 (for PCS and WSAS outcomes)  Qualifications (for PCS and WSAS outcomes)  A stronger conviction in the accuracy of the DS diagnosis (for MCS outcome)  A firm belief in the logic and efficacy of CBT as a treatment (for MCS outcome) | Results of moderator analysis:  Gender: CBT had a positive impact on physical quality of life at 12 months for women as opposed to men  PHQ-15 CBT reduced monthly seizure frequency at 12 months more for participants with a high number of symptoms (≥22) at baseline than for those with fewer symptoms  M.I.N.I. diagnosis:  CBT reduced monthly seizure frequency at 12 months for participants with at least one current M.I.N.I. diagnosis at baseline compared to those without |
| Haug et al. (1994) | functional dyspepsia (symptomatic and endoscopic criteria were applied) | 100 | CBT vs. control group | 10 (50) | Multiple regression analyses | Coefficient of Determination (R^2^),  Regression Coefficient (B),  Standard Error (SE),  95%CI | Dependent Variables:  Dyspeptic symptoms evaluated by endoscopy  Psychological measures:  General Health Questionnaire (GHQ-30)  Spielberger State Trait Anxiety Scale (STAI-I-II)  Beck Depression Inventory (BDI)  Eysenck Personality Questionnaire (EPQ-N)  Sociotropy-Autonomy Scale (SAS)  Dysfunctional Attitude Scale (DAS)  Psychosocial Adjustment to Illness Scale (PAIS)  Giessener Beschwerdebogen  Health Locus of Control Scale (HLCS)  Comprehensive Psychopathological Rating Scale (CPRS) | Treatment arm and target complaints (i.e.,  social, somatic and psychological  problems the patients themselves assumed to be important and wanted to discuss in therapy) | No significant predictor of target complaints between CBT and CG among the assessment tools being applied was found |  |

*Note*. CBT = Cognitive behavioural treatment; CBTZ = Short-Term Psychotherapeutic Intervention (CBT and psychodynamic approach); ECU = Enhanced Care as usual; ENCERT = CBT + emotion regulation training; ET= Exposure therapy; FibroQoL = multicomponent intervention for FM MIBS =Mindfulness for IBS; MBCT = mindfulness-based cognitive therapy; MT = Multidisciplinary treatment; OBT = operant–behavioral therapy; TAU = Treatment as usual; WL = Waiting list

## S2: PICOS criteria for included studies on predictors of treatment success

| Participants: | - Adults with an age of ≥ 18 years - Diagnosis of the following functional disorders:   - Somatoform disorder (including all equivalent terms such as somatization disorder, somatic symptom disorder, bodily distress disorder)   - Irritable bowel syndrome (IBS)   - Chronic fatigue syndrome/myalgic encephalomyelitis (CFS/ME)   - Fibromyalgia - Diagnosis has to refer to a common taxonomy (e.g., ICD, DSM, American College of Rheumatology (ACR) criteria, Rome criteria, Manning criteria, Fukuda definition/Centers for Disease Control and Prevention (CDC) definition, Euro-SOMA) or standardized criteria - Diagnosis by a clinician or a researcher - Use of standardized diagnostic tools or standardized rating scale for assessment   Exclusion:   - Special populations (e.g., veterans, torture victims) |
| --- | --- |
| Interventions: | - Psychotherapy with a minimum of 4 sessions and direct caregiver-patient contact (face to face) in an inpatient or outpatient setting including:   - Cognitive, behavioral and operant treatment approaches such as cognitive behavioral therapy (conventional CBT or third-wave treatments)   - Psychodynamic treatment approaches, e.g., psychoanalysis   - Interpersonal therapy   - Dialectical behavior therapy   - Systemic treatment approaches   - Biofeedback techniques within a psychosocial therapeutic setting - Multicomponent treatment (psychosocial therapeutic intervention, see above) if the main ingredient of the intervention is a psychotherapeutic intervention   Exclusion:   - Pharmacological studies - Studies using a surgical method - Experimental approaches (e.g., dead sea therapy) - Self-help interventions |
| Comparisons: | - Direct comparison of the psychosocial therapies in comparison with   - Waiting lists   - Placebo (attention placebo, psychological placebo, pill placebo)   - TAU   - Enhanced care (e.g., additional psychoeducational interventions)   - Non-directive approaches   - Any other interventions when not fulfilling the above-mentioned exclusion criteria |
| Outcomes: | - Primary outcome: Predictor of treatment success   - Symptom intensity/severity   - Functioning: e.g., quality of life, disability, well-being - Secondary outcome: Predictor of treatment success   - Severity of mental comorbidities (e.g., depression, anxiety, general psychopathology)   - Number of somatic symptoms (e.g., measured by NNT, remission rate, response rate) - Predictors (e.g., standardized mean differences, binary outcomes such as risks and odds ratio) - Primary and secondary outcomes at the end of the treatment will be included in the meta-analysis - If studies report on both, a baseline and follow-up measurements, outcomes will be reported and analyzed separately |
| Study design(s): | - Randomized controlled trials with at least 10 subject per arm/group - Clinical population   Exclusion:  • Quasi-randomized trials  • Cluster randomized controlled trials (CRCTs)  Language restriction: none |

## S3: Search terms for PubMed with subsequent filters: adults > 19 years

| Group 1 | (((((((“Functional disorder”) OR ("functional somatic*")) OR ("functional symptom*")) OR ("functional syndrome*")) OR ("functional symptomatic")) OR ("functional illness*")) OR (Functional limitation*)) OR ("functional disease*") |
| --- | --- |
| Group 2 | ((((((((((((bodily distress) OR (somatoform)) OR (dissociat*)) OR (conversion)) OR (Somatic symptom)) OR (Illness anxiety)) OR (Hypochond*)) OR (somatizer*)) OR (hysteri*)) OR ("somatic symptom and related")) OR (SSD)) OR (Neurasthenia)) OR (briquet) |
| Group 3 | ((((((((((((((((((((Medically unexplained) OR ("organically unexplain*")) OR (psychophysiological disorder)) OR (psychosomatic medicine)) OR (psychosomat*)) OR (psychogen*)) OR ("persistent physical symptom*")) OR ("persistent somatic symptom")) OR ("physical symptom disorder")) OR (multisomat*)) OR (polysymptom*)) OR (MUS)) OR (MUPS)) OR (FSS)) OR ("stress disorder*")) OR ("distress disorder*")) OR ("psychological factors affecting med*")) OR ("unexplained medical*")) OR ("unexplained symptom*")) OR ("multiple physical symptom*")) OR ("multiple symptom* diagnos*") |
| Group 4 | ((((((((((((Irritable bowel syndrome*) OR (“functional gastro-intestinal")) OR ("functional intestinal")) OR (“functional esophageal")) OR ("functional constipation")) OR ("functional dyspepsia")) OR ("functional diarrhea")) OR ("functional urinary")) OR ("functional colonic disease*")) OR (IBS)) OR (heart neurosis)) OR (“functional cardiovascular")) OR ("functional palpitation") |
| Group 5 | ((((((((((((((((persistent pain) OR (chronic pain)) OR (Fibromy*)) OR (tension headache*)) OR ("chronic musculoskeletal pain")) OR ("functional abdominal pain")) OR ("tension type headache")) OR (fibrositis)) OR (fibromyositis)) OR (myofibrositis)) OR ("chronic widespread pain")) OR ("widespread musculoskeletal pain")) OR ("myofascial pain")) OR ("chronic intractable benign pain*")) OR (non cardiac chest pain)) OR (non specific chest pain)) OR (atypical pain) |
| Group 6 | (((((((((((((((((Sensory disorder) OR (functional neurological)) OR ("functional movement")) OR ("functional epileptic seizures")) OR ("dissociative seizures")) OR ("psychogenic epileptic seizure*")) OR (Functional cognitive disorder*)) OR ("functional dizziness")) OR ("Functional Paresthesia")) OR ("psychogenic pruritus")) OR (non-epileptic seizures)) OR (chronic fatigue syndrome*)) OR ("Myalgic encephalomyelitis")) OR ("psychogenic fatigue")) OR ("myalgic encephalomyelitis*")) OR ("myalgic encephalopath*")) OR (CFS)) OR ("CFS/ME") |
| Group 7 | “psychotherapy” |
| Group 8 | efficacy OR effective* OR benefit OR predict*” |
| Group 9 | ((RCT) OR ("randomized controlled trial*")) OR ("randomised controlled trial*")) |
| Results: | (#1 OR #2 OR #3 OR #4 OR #5) AND #7 AND #8 AND #9 |

# References

Abbass, A., M. A. Lumley, J. Town, H. Holmes, P. Luyten, A. Cooper, L. Russell, H. Schubiner, C. De Meulemeester and S. Kisely (2021). "Short-term psychodynamic psychotherapy for functional somatic disorders: A systematic review and meta-analysis of within-treatment effects." Journal of Psychosomatic Research **145**: 110473.

American Psychiatric Association, A. and A. P. Association (1994). Diagnostic and statistical manual of mental disorders: DSM-IV, American psychiatric association Washington, DC.

Amris, K., G. Luta, R. Christensen, B. Danneskiold-Samsøe, H. Bliddal and E. E. Wæhrens (2016). "Predictors of improvement in observed functional ability in patients with fibromyalgia as an outcome of rehabilitation." Journal of Rehabilitation Medicine **48**(1): 65-71.

Burton, C., P. Fink, P. Henningsen, B. Löwe and W. Rief (2020). "Functional somatic disorders: discussion paper for a new common classification for research and clinical use." Bmc Medicine **18**(1): 1-7.

Carle-Toulemonde, G., J. Goutte, N. Do-Quang-Cantagrel, S. Mouchabac, C. Joly and B. Garcin (2023). "Overall comorbidities in functional neurological disorder: A narrative review." L'encephale.

Caroline Rometsch, G. M., Sara Romanazzo, Alexandra Martin, Fiammetta Cosci (2021). "Transdiagnostic prevalence of functional disorders across Europe: A systematic literature review and meta-analysis." PROSPERO: CRD42022298974

Chaabouni, A., J. Houwen, I. Walraven, K. van Boven, H. Peters, H. Schers and T. olde Hartman (2023). "Patients’ characteristics and general practitioners’ management of patients with symptom diagnoses." The Journal of the American Board of Family Medicine **36**(3): 477-492.

Chowdhury, M. Z. I. and T. C. Turin (2020). "Variable selection strategies and its importance in clinical prediction modelling." Family medicine and community health **8**(1).

Dale, R., K. Limburg, G. Schmid-Mühlbauer, T. Probst and C. Lahmann (2023). "Somatic symptom distress and gender moderate the effect of integrative group psychotherapy for functional vertigo on vertigo handicap: A moderation analysis of a randomised controlled trial." Journal of Psychosomatic Research **167**: 111175.

De Waal, M. W. M., I. A. Arnold, J. A. Eekhof and A. M. Van Hemert (2004). "Somatoform disorders in general practice: prevalence, functional impairment and comorbidity with anxiety and depressive disorders." The British Journal of Psychiatry **184**(6): 470-476.

Drossman, D. A. and W. G. Thompson (1992). "The irritable bowel syndrome: review and a graduated multicomponent treatment approach." Annals of Internal Medicine **116**(12_Part_1): 1009-1016.

Engel, G. L. (1977). "The need for a new medical model: a challenge for biomedicine." Science **196**(4286): 129-136.

Fava, G. A. (2011). "The clinical factor." Psychotherapy and Psychosomatics **80**(1): 1-3.

Fava, G. A., F. Cosci and N. Sonino (2017). "Current psychosomatic practice." Psychotherapy and psychosomatics **86**(1): 13-30.

Fava, G. A., H. J. Freyberger, P. Bech, G. Christodoulou, T. Sensky, T. Theorell and T. N. Wise (1995). "Diagnostic criteria for use in psychosomatic research." Psychotherapy and psychosomatics.

Flückiger, C., B. E. Wampold, J. Delgadillo, J. Rubel, A. Vîslă and W. Lutz (2020). "Is there an evidence-based number of sessions in outpatient psychotherapy?–A comparison of naturalistic conditions across countries." Psychotherapy and psychosomatics **89**(5): 333-335.

Friedman, L. M., C. D. Furberg, D. L. DeMets, D. M. Reboussin and C. B. Granger (2015). Fundamentals of clinical trials, Springer.

Gergov, V., N. Lindberg, J. Lahti, J. Lipsanen and M. Marttunen (2021). "Effectiveness and predictors of outcome for psychotherapeutic interventions in clinical settings among adolescents." Frontiers in Psychology **12**: 628977.

Goldstein, L., E. Robinson, T. Chalder, J. Stone, M. Reuber, N. Medford, A. Carson, M. Moore and S. Landau (2022). "Moderators of cognitive behavioural therapy treatment effects and predictors of outcome in the CODES randomised controlled trial for adults with dissociative seizures." Journal of Psychosomatic Research **158**: 110921.

Guthrie, E., F. Creed, D. Dawson and B. Tomenson (1991). "A controlled trial of psychological treatment for the irritable bowel syndrome." Gastroenterology **100**(2): 450-457.

Haug, T. T., I. Wilhelmsen, S. Svebak, A. Berstad and H. Ursin (1994). "Psychotherapy in functional dyspepsia." Journal of Psychosomatic Research **38**(7): 735-744.

Häuser, W., K. Bernardy, B. Arnold, M. Offenbächer and M. Schiltenwolf (2009). "Efficacy of multicomponent treatment in fibromyalgia syndrome: a meta‐analysis of randomized controlled clinical trials." Arthritis Care & Research **61**(2): 216-224.

Henrich, J. F., B. Gjelsvik, C. Surawy, E. Evans and M. Martin (2020). "A randomized clinical trial of mindfulness-based cognitive therapy for women with irritable bowel syndrome—Effects and mechanisms." Journal of Consulting and Clinical Psychology **88**(4): 295.

Hobson, R. (1985). Forms of Feeling., London, Tavistock Publications.

Kleinstaeuber, M., M. Witthoeft, A. Steffanowski, H. van Marwijk, W. Hiller and M. J. Lambert (2014). "Pharmacological interventions for somatoform disorders in adults." Cochrane Database of Systematic Reviews(11).

Kohlmann, S., B. Löwe and M. C. Shedden-Mora (2018). "Health care for persistent somatic symptoms across Europe: a qualitative evaluation of the EURONET-SOMA expert discussion." Frontiers in psychiatry **9**: 646.

Kolk, A., S. Schagen and G. Hanewald (2004). "Multiple medically unexplained physical symptoms and health care utilization: outcome of psychological intervention and patient-related predictors of change." Journal of psychosomatic research **57**(4): 379-389.

Kustra-Mulder, A., B. Löwe and A. Weigel (2023). "Healthcare-related factors influencing symptom persistence, deterioration, or improvement in patients with persistent somatic symptoms: A scoping review of European studies." Journal of Psychosomatic Research: 111485.

Lagrand, T. J., M. Jones, A. Bernard and A. C. Lehn (2023). "Health Care Utilization in Functional Neurologic Disorders: Impact of Explaining the Diagnosis of Functional Seizures on Health Care Costs." Neurology: Clinical Practice **13**(1).

Lee, Y., R.-M. Ragguett, R. B. Mansur, J. J. Boutilier, J. D. Rosenblat, A. Trevizol, E. Brietzke, K. Lin, Z. Pan and M. Subramaniapillai (2018). "Applications of machine learning algorithms to predict therapeutic outcomes in depression: A meta-analysis and systematic review." Journal of affective disorders **241**: 519-532.

Lera, S., S. M. Gelman, M. J. López, M. Abenoza, J. G. Zorrilla, J. Castro-Fornieles and M. Salamero (2009). "Multidisciplinary treatment of fibromyalgia: does cognitive behavior therapy increase the response to treatment?" Journal of psychosomatic research **67**(5): 433-441.

Liu, J., N. S. Gill, A. Teodorczuk, Z.-j. Li and J. Sun (2019). "The efficacy of cognitive behavioural therapy in somatoform disorders and medically unexplained physical symptoms: A meta-analysis of randomized controlled trials." Journal of affective disorders **245**: 98-112.

Ljótsson, B., G. Andersson, E. Andersson, E. Hedman, P. Lindfors, S. Andréewitch, C. Rück and N. Lindefors (2011). "Acceptability, effectiveness, and cost-effectiveness of internet-based exposure treatment for irritable bowel syndrome in a clinical sample: a randomized controlled trial." BMC gastroenterology **11**(1): 1-13.

Löwe, B. and C. Gerloff (2018). "Functional somatic symptoms across cultures: perceptual and health care issues." Psychosomatic medicine **80**(5): 412-415.

Mamo, N., M. van de Klundert, L. Tak, T. O. Hartman, D. Hanssen and J. Rosmalen (2023). "Characteristics of collaborative care networks in functional disorders: A systematic review." Journal of Psychosomatic Research: 111357.

Maroti, D., M. A. Lumley, H. Schubiner, P. Lilliengren, I. Bileviciute-Ljungar, B. Ljótsson and R. Johansson (2022). "Internet-based emotional awareness and expression therapy for somatic symptom disorder: A randomized controlled trial." Journal of Psychosomatic Research **163**: 111068.

McDonald, S., M. Melkonian, E. Karin, B. F. Dear, N. Titov and B. M. Wootton (2023). "Predictors of response to cognitive behavioural therapy (CBT) for individuals with obsessive-compulsive disorder (OCD): a systematic review." Behavioural and Cognitive Psychotherapy: 1-18.

Melidis, C., S. L. Denham and M. E. Hyland (2018). "A test of the adaptive network explanation of functional disorders using a machine learning analysis of symptoms." Biosystems **165**: 22-30.

Methley, A. M., S. Campbell, C. Chew-Graham, R. McNally and S. Cheraghi-Sohi (2014). "PICO, PICOS and SPIDER: a comparison study of specificity and sensitivity in three search tools for qualitative systematic reviews." BMC health services research **14**(1): 1-10.

Pérez-Aranda, A., A. Feliu-Soler, J. Montero-Marín, J. García-Campayo, L. Andrés-Rodríguez, X. Borràs, A. Rozadilla-Sacanell, M. T. Peñarrubia-Maria, N. Angarita-Osorio and L. M. McCracken (2019). "A randomized controlled efficacy trial of mindfulness-based stress reduction compared with an active control group and usual care for fibromyalgia: The EUDAIMON study." Pain **160**(11): 2508-2523.

Richtberg, S., M. Jakob, V. Höfling and F. Weck (2017). "Patient characteristics and patient behavior as predictors of outcome in cognitive therapy and exposure therapy for hypochondriasis." Journal of clinical psychology **73**(6): 612-625.

Rosmalen, J., C. Burton, A. Carson, F. Cosci, L. Frostholm, N. Lehnen and T. O. Hartman (2021). "The European Training Network ETUDE (Encompassing Training in Functional Disorders across Europe) Is Recruiting 15 Early-Stage Researchers." Psychotherapy and psychosomatics **90**(2): 142-144.

Rosmalen, J., C. Burton, A. Carson, F. Cosci, L. Frostholm, N. Lehnen, T. Olde Hartman, C. Rask, J. Rymaszewska and J. Stone (2021). "The European Training Network ETUDE (Encompassing Training in fUnctional Disorders across Europe): a new research and training program of the EURONET-SOMA network recruiting 15 early stage researchers."

Sajjadian, M., R. W. Lam, R. Milev, S. Rotzinger, B. N. Frey, C. N. Soares, S. V. Parikh, J. A. Foster, G. Turecki and D. J. Müller (2021). "Machine learning in the prediction of depression treatment outcomes: a systematic review and meta-analysis." Psychological Medicine **51**(16): 2742-2751.

Sarter, L., J. Heider, M. Witthöft, W. Rief and M. Kleinstäuber (2022). "Using clinical patient characteristics to predict treatment outcome of cognitive behavior therapies for individuals with medically unexplained symptoms: A systematic review and meta-analysis." General hospital psychiatry **77**: 11-20.

Saunders, C., H. Treufeldt, M. T. Rask, H. F. Pedersen, C. Rask, C. Burton and L. Frostholm (2023). "Explanations for functional somatic symptoms across European treatment settings: A mixed methods study." Journal of Psychosomatic Research **166**: 111155.

Schmidt, T., G. Ebersbach, H. Oelsner, A. Sprock, I. R. König, T. Bäumer, A. Münchau and A. Weissbach (2021). "Evaluation of Individualized Multi‐Disciplinary Inpatient Treatment for Functional Movement Disorders." Movement Disorders Clinical Practice **8**(6): 911-918.

Senger, K., A. Schröder, M. Kleinstäuber, J. A. Rubel, W. Rief and J. Heider (2022). "Predicting optimal treatment outcomes using the Personalized Advantage Index for patients with persistent somatic symptoms." Psychotherapy Research **32**(2): 165-178.

Tanguay-Sela, M., C. Rollins, T. Perez, V. Qiang, G. Golden, J.-F. Tunteng, K. Perlman, J. Simard, D. Benrimoh and H. C. Margolese (2022). "A systematic meta-review of patient-level predictors of psychological therapy outcome in major depressive disorder." Journal of Affective Disorders.

Thieme, K., D. C. Turk and H. Flor (2007). "Responder criteria for operant and cognitive–behavioral treatment of fibromyalgia syndrome." Arthritis Care & Research: Official Journal of the American College of Rheumatology **57**(5): 830-836.

Tummers, M., H. Knoop and G. Bleijenberg (2010). "Effectiveness of stepped care for chronic fatigue syndrome: a randomized noninferiority trial." Journal of Consulting and Clinical Psychology **78**(5): 724.

Vallejo, M. A., J. Ortega, J. Rivera, M. I. Comeche and L. Vallejo-Slocker (2015). "Internet versus face-to-face group cognitive-behavioral therapy for fibromyalgia: a randomized control trial." Journal of Psychiatric Research **68**: 106-113.

Van Ravesteijn, H., P. Lucassen, H. Bor, C. Van Weel and A. Speckens (2013). "Mindfulness-based cognitive therapy for patients with medically unexplained symptoms: a randomized controlled trial." Psychotherapy and Psychosomatics **82**(5): 299-310.

Weck, F., J. M. Neng, J. Schwind and V. Höfling (2015). "Exposure therapy changes dysfunctional evaluations of somatic symptoms in patients with hypochondriasis (health anxiety). A randomized controlled trial." Journal of Anxiety Disorders **34**: 1-7.
